# Monte Carlo analysis of an ODE Model of the Sea Urchin Endomesoderm Network

**DOI:** 10.1186/1752-0509-3-83

**Published:** 2009-08-23

**Authors:** Clemens Kühn, Christoph Wierling, Alexander Kühn, Edda Klipp, Georgia Panopoulou, Hans Lehrach, Albert J Poustka

**Affiliations:** 1 Max-Planck-Institute for Molecular Genetics, Ihnestr 63-73, 14195 Berlin, Germany; 2 Humboldt Universität zu Berlin, Institute for Biology, Invalidenstr 42, 10115 Berlin, Germany

## Abstract

**Background:**

Gene Regulatory Networks (GRNs) control the differentiation, specification and function of cells at the genomic level. The levels of interactions within large GRNs are of enormous depth and complexity. Details about many GRNs are emerging, but in most cases it is unknown to what extent they control a given process, i.e. the grade of completeness is uncertain. This uncertainty stems from limited experimental data, which is the main bottleneck for creating detailed dynamical models of cellular processes. Parameter estimation for each node is often infeasible for very large GRNs. We propose a method, based on random parameter estimations through Monte-Carlo simulations to measure completeness grades of GRNs.

**Results:**

We developed a heuristic to assess the completeness of large GRNs, using ODE simulations under different conditions and randomly sampled parameter sets to detect parameter-invariant effects of perturbations. To test this heuristic, we constructed the first ODE model of the whole sea urchin endomesoderm GRN, one of the best studied large GRNs. We find that nearly 48% of the parameter-invariant effects correspond with experimental data, which is 65% of the expected optimal agreement obtained from a submodel for which kinetic parameters were estimated and used for simulations. Randomized versions of the model reproduce only 23.5% of the experimental data.

**Conclusion:**

The method described in this paper enables an evaluation of network topologies of GRNs without requiring any parameter values. The benefit of this method is exemplified in the first mathematical analysis of the complete Endomesoderm Network Model. The predictions we provide deliver candidate nodes in the network that are likely to be erroneous or miss unknown connections, which may need additional experiments to improve the network topology. This mathematical model can serve as a scaffold for detailed and more realistic models. We propose that our method can be used to assess a completeness grade of any GRN. This could be especially useful for GRNs involved in human diseases, where often the amount of connectivity is unknown and/or many genes/interactions are missing.

## Background

Today, experimental research has uncovered a great amount of regulatory interactions between different transcription factors (TFs). These interactions can be summarized in Gene Regulatory Networks (GRNs) that control the differentiation, specification and function of cells at the genomic level. The levels of interactions within large GRNs are of enormous depth and complexity. Details about many GRNs are emerging, but in most cases it is unknown to what extent they properly describe a given process, i.e. the grade of completeness is uncertain. This uncertainty stems from limited experimental data, which is the main bottleneck for creating detailed dynamical models of cellular processes. Parameter estimation for each node is often infeasible for very large GRNs. These GRNs are static representations of the interactions and can provide scaffolds for fine grained low-level models [1]. A mathematical low-level model allows for a detailed quantitative analysis of the system and has predictive power. Construction of detailed quantitative models of large GRNs is often infeasible because the underlying data is too sparse to parameterize the model. Analysis of model properties on static network graphs, on the other hand, provides only limited insights.

To circumvent these shortcomings, we propose to construct a scaffold model of ordinary differential equations (ODEs). Analysis of some key properties of this model is feasible without knowledge of the kinetic parameters. The detected properties are compared to experimental data for validation. Parameterization of this model can be achieved by iteratively improving parts of the model once sufficient data become available.

**Figure 1:**
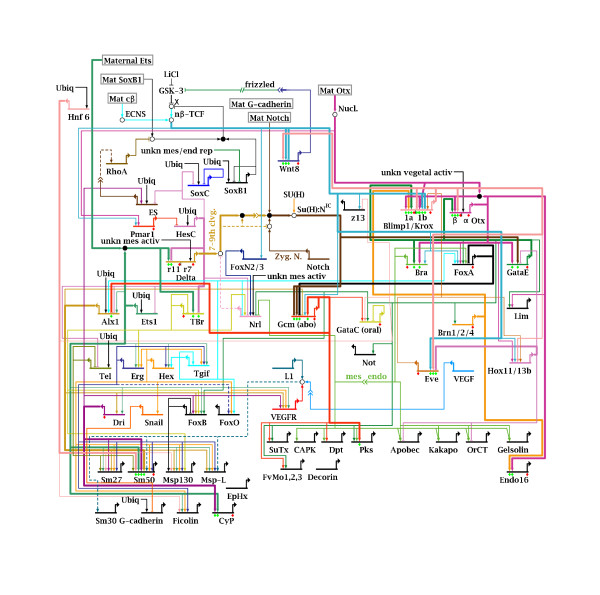
**Topology of the Endomesoderm Network**. The Endomesoderm GRN, as used in this analysis. Reproduced from [4] (version of December 2007) with permission from E.H. Davidson. Horizontal lines with bent outward arrow indicate genes. Arrows towards genes indicate activators of transcription, barred lines incident to genes indicate inhibitors of expression. Each gene is assumed to be exclusively regulated by the interactions shown. Protein interactions are indicated by circles. For details of the model, please refer to Additional Files 1 and 6. Genes or proteins without any input shown were set manually in simulations. Cis-regulatory inputs to genes that have not been identified by experimental analysis are denoted 'Ubiq', 'Mat' denotes amternal inputs, 'nucl.' indicates nuclearization and 'X' refers to the source of *β*-catenin. The different territories simulated contain the same set of possible interactions but due to temporally differing inputs, different subsets are realized in each territory.

As an example application for our approach, we construct a provisional scaffold model for gene regulation in the early sea urchin embryo based on the Endomesoderm GRN, one of the best studied large developmental GRNs. The Endomesoderm Gene Regulatory Network provides the genetic hardwiring of the control and regulation of gene expression during development of the endoderm, mesoderm and primary mesenchyme cells (PMC) [2]. These territories mainly arise from the macromeres (endoderm and mesoderm) and micromeres (mesoderm and PMC). For further details on the embryogenesis of the sea urchin, please refer to [3]. The endomesoderm GRN (Figure 1 and Additional file 6) describes the regulation of the expression of 60 genes (as of December 2007) as well as intercellular signaling (Delta/Notch) and protein interactions (Wnt-Pathway). The network is constantly updated, thus the actual number of genes and topology described at [4] now differs from that used here.

The structure of the network is constructed from a multitude of perturbation experiments, including injection of morpholino-substituted antisense oligonucleotides that effectively knock down mRNA translation (KD) of a specific gene [5], injection of engrailed repressor domain fusions to turn activators into repressors, or mRNA over expression (MOE). The network as described in [4] is an interpretation of a vast amount of experimental data. As pointed out by Smith [6], perturbation experiments may lead to ambiguous results, which need to be clarified in subsequent experiments. Although the experimental results currently available are solid and only error prone due to incompleteness, we believe that a computational analysis of the Endomesoderm Network could greatly substantiate its interpretations. Additionally, the analysis of a mathematical model allows for the detection of possible errors and identification of ambiguities in a more comprehensive way.

**Figure 2:**
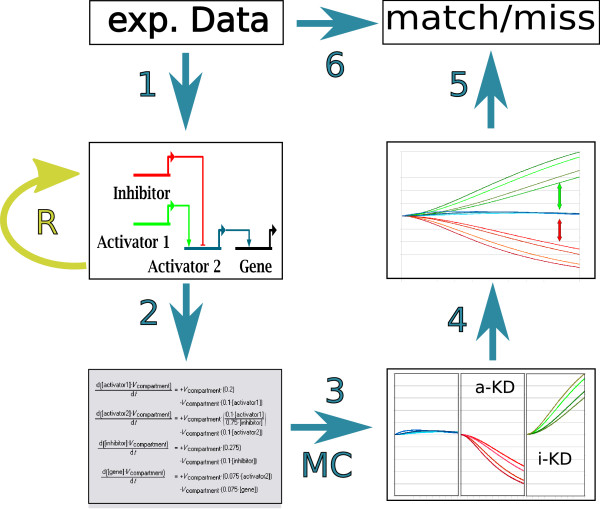
**Schematic summary of the detection of TPEs**. The Endomesoderm GRN was derived from experimental data (step 1), from this topology we derive an ODE model (2) which is then repeatedly simulated with randomly sampled parameter sets under different perturbations (3). Exemplary, the possible effect of knock down of an activator (a-KD) and an inhibitor (i-KD) on a genes expression are shown. Qualitative topological perturbation effects, the sign of the difference between control and a-KD resp. i-KD indicated by the red and green arrows, are detected from these simulations (4) and these effects compared to experimental data (5). For the comparison, quantitative experimental data needs to be transformed to qualitative data (6). In order to rank the agreement between network topology and experimental data, the same method is applied to randomized networks (*R*). Alternatively, the resulting agreement can be compared to the agreement expected given that the correct topology is used, as described in section 'Inference of the Reliability of the Proposed Heuristic' and sketched in Figure 3.

Since the underlying data are mainly the results of perturbation experiments and time course data for mRNA abundances are available for only a small subset of genes ([7-14] at the start of our study in December 2007), this model cannot be fully parameterized from experimental data. Parameter estimation is also impractical due to the size of the model. The experimental data lack sufficient detail to unambiguously assign realistic kinetics for transcriptional activation and repression to each gene in the network. We therefore chose a generalized approach for the construction of transcription kinetics. In order to analyze our ODE model with regard to the perturbation data, we developed a Monte Carlo method to assess the qualitative effects of perturbations (here knockdown or over expression of a gene) of an ODE model in spite of sparse data. A schematic description of this method is given in Figure 2. The method uses randomly sampled sets of parameters to detect parameter-invariant, qualitative perturbation effects. For each set of parameters, we simulate the model under different experimental conditions. Comparison of these different simulations reveals downstream effects of applied perturbations, which persist over a large range of parameter sets. These effects are compared to experimental data, indicating to what extent the model topology qualitatively reproduces the experimental data. Although developmental systems have been shown to exhibit a high degree of robustness to genetic variation [15], one cannot assume that all interactions within a developmental GRN are invariant to parameter changes. The activity of negative and positive feedback loops, for example, depends on quantitative information. This holds true for the Endomesoderm Network, so we do not expect our model to reproduce the experimental data perfectly, even if the underlying topology is completely accurate. To rank the agreement between simulation and experimental results, we repeat the analysis with randomized network topologies.

The detailed results of our analysis show which parts of the model reproduce the experimental data exceptionally well or exceptionally poor. Additionally, our analysis shows a large number of effects not yet tested experimentally. These predictions can be used for experimental validation.

Our work provides a scaffold for quantitative kinetic modeling of the endomesoderm network, publicly available in SBML format in the BioModels database [16]. Refinement of this scaffold model is straightforward: Well studied parts of the model (e.g. the PMC region) can be refined in isolation and then reintegrated upon the scaffold. Such a stepwise improvement of the model is, in our opinion, less error prone than setting up a comprehensive detailed model from scratch. The iterative nature of the approach also provides a simple way to organize concerted efforts and directly shows which parts of the endomesoderm GRN require most attention in future experimental research.

## Results and discussion

### Heuristic Inference of Topological Perturbation Effects of ODE Models

In the following, we describe the method we use to detect topological perturbation effects from ODE models of GRNs and give a sample application that indicates the reliability of this method. The heuristic we propose here allows for an assessment of the correctness of a GRN topology given the underlying data without the need to estimate parameter values. It is thus applicable to very large GRNs. A diagram of the method is given in Figure 2.

If realistic kinetic parameters for a given GRN model are known, the quantitative effect of the knockdown (KD) or mRNA overexpression (MOE) of one or more of its components can be simulated. The qualitative effect of KD or MOE conditions that are invariant to the choice of parameters can be computed for large networks using our method. Since these effects are inherent in the topology of the GRN, we call them topological perturbation effects (TPE).

Extraction of TPEs from small GRNs can be straightforward and obvious, but for large GRNs, manual detection of all topological features becomes infeasible and error-prone. To detect TPEs of a given network topology, logical models can be applied [17]. The advantage of our continuous approach is that the model can be improved stepwise by restricting the distribution from which the parameters are drawn or fixing certain known parameters, eventually yielding a comprehensible quantitative model.

Given the topology of a GRN that has been constructed in some way from experimental data (step 1 in Figure 2), we construct a corresponding ODE model *M*_0 _(step 2 in Figure 2). For each experimental perturbation *Pert*_*i*_, a model variant *M*_*i *_is created and all model variants are simulated using different randomly sampled parameter sets *P*_1..*K*_, yielding simulation results  (step 3 in Figure 2). Differences in simulation results for a pair  that are consistent under most parameter sets are detected as TPEs (step 4 in Figure 2). The effects detected with this Monte Carlo approach are then compared to experimental data to compute the agreement between experimental data and constructed model (step 5 in Figure 2).

TPEs can be detected for a wide range of perturbations to the model. In contrast to experimental cis-regulatory analysis, this computational approach can identify the effects of all possible perturbations on all genes in a proposed GRN simultaneously. This predictive power of the analysis can be used to test the proposed GRN by targeted experimental research.

### Inference of the Reliability of the Proposed Heuristic

**Figure 3:**
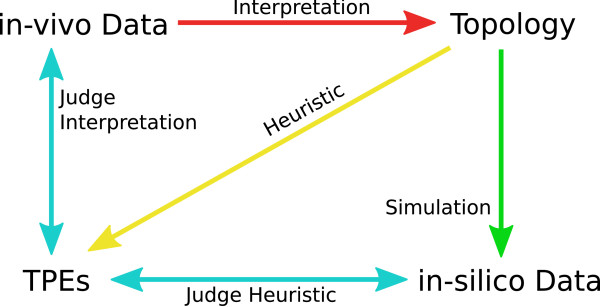
**Validation of our Method**. Schematic representation of the approach we use to evaluate the heuristic we propose. The topology of a given GRN is inferred from *in-vivo *data (red arrow). To judge this step, we propose our heuristic as described in text and Figure 2 (yellow arrow). The comparison of *in-vivo *data and detected TPEs yields some measure of matching effects (left light blue arrow). In order to evaluate this, we simulate *in-silico *data that perfectly matches an underlying topology (green arrow). Comparison between *in-silico *data and detected TPEs indicates the reliability of the heuristic (lower light blue arrow).

The agreement between TPEs and *in-vivo *data depends on the reliability of our heuristic. Assume that our heuristic is expected to return an agreement of 80% if a given topology inferred from experimental data is correct. If we then test another topology obtained from a different set of data and get an agreement of 75%, the network is close to completeness because 75% of the optimal 80% are 94%. To obtain a correct topology and underlying data to determine the reliability of our heuristic, we used *in-silico *data simulated from a given topology. This approach is sketched in Figure 3. We chose a subnetwork of the endomesoderm network [18] for this test.

The chosen subnetwork consists of interactions between *Blimp1*, *Bra*, *Brn*, *Eve*, *FoxA*, *GataE*, *Hox*, *Otx*, *Pmar1 *and *Wnt8*. Parameters have been estimated for this model [18]. We denote the *in-silico *perturbation data (*KM*^*est*^) and the computed TPEs (*KM*^*mc*^).

Comparison of the *in-silico *data *KM*^*est*^, which contains TPEs and parameter-specific effects, and TPEs *KM*^*mc*^show the following results: 73.8% of the perturbation effects were equally detected in both settings. False negatives (effects detected using *KM*^*mc *^and not detected using *KM*^*est*^) constitute 8.1%, false positives constitute 15.7% and 2.1% of the effects are detected with opposite signs in the two settings. This indicates that our heuristic approach to identify TPEs yields satisfying qualitative results at least for small networks. If the wiring of the model is equal to the real topology, about 74% of TPEs detected with our Monte Carlo approach should match experimental perturbation data.

### Assumptions Underlying the ODE Model of the Endomesoderm GRN

The method explained above is most useful when dealing with large models associated with sparse experimental data. Parameter estimation is infeasible here, but our method allows a preliminary assessment of the main features and comparison to experimental data. The endomesoderm network is a large network with sparse associated data. In order to set up the actual ODE model, we make the following assumptions concerning the relevant biological mechanisms during development and their mathematical representation. The model is concerned with the endoderm, mesoderm and PMC territories of the early sea urchin embryo. For detailed information on sea urchin development, please refer to [2] and references therein.

Although each embryonic territory differs concerning the expression of genes, abundance of transcription factors (TF) and signaling molecules, we assume that each cell contains the same genetic information, i.e. that no histone modification occurs in the early stages of development. We assume that each territory consists of a homogeneous number of cells, i.e. that cells in the same territory contain the same combination of TFs and express the same genes. If gene expression is regulated in part by extracellular gradients, these regulating gradients can be assumed to differ throughout individual territories and thus the regulated genes are also likely to be expressed differently in different parts of the same territory. But unless we simulate a whole population of cells (see [19-21] for approaches to modeling populations of cells), an ODE model is only capable of assessing averages of this population.

In our model, we will not try to reproduce expression time courses or spatial expression patterns. The objective is to detect the topological features arising from the Endomesoderm GRN and to compare them with experimental perturbation data. The assumptions stated above enable a straightforward modeling of the three territories referenced in the Endomesoderm Network: each territory (endoderm, mesoderm and PMC) is modeled as one cell, each containing the same regulatory interactions.

Important signaling events in development are controlled by intercellular signaling, either direct or by extracellular gradients. The effects of intercellular signaling are necessary to produce differential expression, one of the key aspects of development. For simplicity, we reduce the dynamic interaction between the different cells of the embryo to a static input depending on assigned cell type and simulation time. Making this simplification, we lose possible topological effects that manifest only in the interaction between cells. On the other hand, we avoid possible errors as well as excessive computation. Realistic modeling of extracellular gradients would require spatial modeling of a growing embryo. This would require simulation of a growing population of cells each containing an ODE model. The population of cells would need to contain territories not included in the Endomesoderm Network. It would further require realistic numbers of cells in each territory and subpopulation of a territory producing a specific extracellular signal and additional assumptions concerning diffusion rates.

An ODE model constructed according to the assumptions made above can produce differential expression in different territories depending on the static inputs. It can simulate the effect of perturbations within a territory. It cannot simulate the effects of perturbations that propagate through intercellular signaling unless the static inputs are modified to dynamic interactions between the different territories.

### Construction of the ODE Model of the Endomesoderm Network

The assumptions made above are used to construct an ODE model from the topology of the Endomesoderm Network (Step2 in Figure 2). Since the detailed cis-regulatory interactions, and thus transcription kinetics, are not available for all genes in the network, we chose to construct a provisional scaffold model including all genes, but using simplified transcription kinetics. From this provisional model, we hope to eventually set up a realistic model, refining individual transcription kinetics by hand. For most genes in the network, the regulatory inputs and their nature are known. But the detailed cis-regulatory wiring, which would be necessary to set up realistic transcription kinetics, is only known for a very limited number of genes, for example *Endo16 *[22]. Therefore, we chose the following simplified approach.

**Figure 4:**
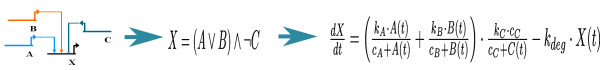
**Construction of ODEs from network topology**. Schematic representation of the conversion of a GRN topology to an ODE model. In the diagram to the left, *A*, *B*, *C *and *X *denote genes. In the Boolean formula in the center, the variables represent the gene products, translation and transcription are combined to one step. In the differential equation to the right, *X *indicates concentration of mRNA of gene *X*, while *A*, *B *and *C *are protein concentrations, small letters are kinetic parameters. The topology is used to derive a Boolean formulation of the regulatory inputs to a gene. This Boolean formulation is automatically translated to an ODE model. For details, see section Methods.

To construct an ODE model, the Endomesoderm Network (Figure 1 and Additional file 6) is first transformed into a Boolean model. We used the data included in the Biotapestry [23] view of the network (as of December 2007) to determine the kind of regulatory interaction between a TF and the expression of a gene as well as the Boolean operator used to combine different inputs to one gene. In general, two activating inputs were connected using the OR operator, inhibiting inputs were appended using the AND operator. The Boolean model has 169 gene regulatory interactions, of which 131 are activating and 38 are inhibiting. In total, the model contains 38 AND and 94 OR operators. The Boolean formulas for gene regulation are transformed into rate laws for the ODE model (see section Methods and Figure 4). The ODE model also contains translation, mRNA as well as protein degradation and protein interactions, all following mass action kinetics.

The model contains three different embryonic territories, endoderm, mesoderm and primary mesenchyme. The three different territories are identical copies of the transformed Boolean model. External inputs to the different territories are modeled as Hill-functions modulated by events that are territory-specific. Initial conditions were set according to the Biotapestry view of the Endomesoderm Network and are also tissue specific. The model contains 54 genes and 140 variable species, 278 reactions and 287 parameters for each cell type (See Additional File 1 for the rate laws used in the model.).

**Figure 5:**
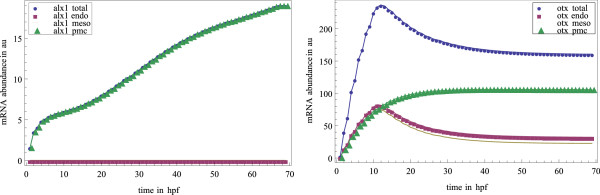
**Simulation of differential expression**. Timecourse simulation for *Alx1 *(left) and *Otx *(right) expression in different territories up to 70 hours post fertilization (hpf) in arbitrary units (au). Parameters of the ODE model are not fitted to reproduce experimental data. Spatial expression patterns are nevertheless generated in our model: Expression in the total embryo (blue) is the sum of expression in endoderm (purple), mesoderm (brown) and PMC (green) expression. The plotted values are the means of 800 simulations using randomly sampled parameter sets (error bars not shown). In the left part, total *Alx1 *mRNA abundance equals abundance in PMC; abundance in endoderm and mesoderm are 0. The simulation results show differential expression in the different territories.

We evaluate the model with respect to qualitative experimental data on perturbation effects, not with respect to quantitative time courses of mRNA abundance. Nevertheless, the constructed model must be capable of producing differential expression in the different territories to qualify as a model of development. Figure 5 shows simulated time courses for different genes and territories using an average of simulation results from different parameter sets. These time courses do not reproduce experimental time courses, but they demonstrate that our provisional model is capable of producing differential expression. The time course for *Alx1*-mRNA abundance clearly shows that it is only expressed under PMC conditions, which agrees with experimental data. *Otx *is expressed in all three territories but to different extents in each territory. The strength of expression in each territory differs due to upstream inputs.

### Monte Carlo simulation of knockdown and overexpression experiments using randomly sampled parameter sets

In order to compare the effects of knockdown (KD) or mRNA overexpression (MOE) perturbations on our model with experimental results, we simulated the unperturbed system and different perturbation experiments (step 3 in Figure 2). To create models of knockdown and overexpression experiments efficiently, we changed the original rate laws for transcription in the following way:

(1)

(2)

(3)

We chose a multiplicative decrease for knockdown experiments because in the analogous experiments, the transcribed mRNA is blocked from translation similarly in case of high and low expression rates.

Overexpression in experiments was brought about by injection of synthetic mRNA, so the resulting increase in expression is independent of the endogenous expression. For this reason we chose an additive increase for overexpression.

**Figure 6:**
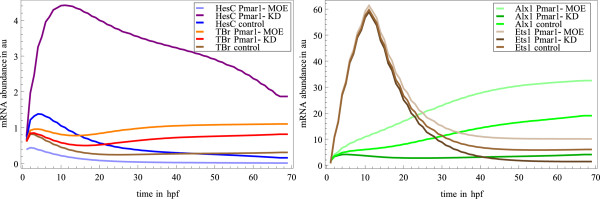
**Effects of perturbations in Pmar1 expression show effects of double-negative regulation of TBr, Alx1 and Ets1**. Simulated timecourse of *HesC*, *TBr *(left), and *Alx1*, *Ets1 *(right) abundance under *Pmar1*-MOE, *Pmar1*-KD and control conditions in arbitrary units up to 70 hours post fertilization. The values plotted are the means of 800 simulation results using different parameter sets (error bars not shown). *HesC*, the only direct target of *Pmar1 *is inhibited by *Pmar1*. The inhibitory role of *HesC *on *TBr*, *Ets1 *and *Alx1 *causes these genes to react as if activated by *Pmar1*.

We simulated all perturbation experiments affecting single genes mentioned in the Endomesoderm QPCR tables [24] as of December, 2007, except for fusion of genes with the engrailed repressor domain. Simulation of these fusion experiments would have required careful adjustments of all affected rate laws and would have required additional assumptions on the mechanisms of the fused TFs in combination with other TFs. The set *M*_1..*N *_(see section Methods for details) of perturbed models contains *Alx1 *KD, *Alx1 *MOE, *Pmar1 *KD, *Pmar1 *MOE, *SoxB1 *KD, *SoxB1 *MOE, *Dri *KD, *Ets1 *KD, *Hnf6 *KD, *TBr *KD, *Snail *KD, *FoxB *KD, *Brn *KD, *Eve *KD, *Blimp1 *KD, *GataE *KD, *Hox *KD, *Gcm *KD, *FoxA *KD, *Bra *KD, *Otx *KD, *GataC *KD. We simulated both the original and the perturbed models using 800 parameter sets. As an example, we present the effects of *Pmar1 *perturbations on several genes, *Alx1*, *Ets1*, *Tbr *and *HesC*, in detail. The means of the mRNA abundance over all parameter sets for different perturbation conditions are plotted as time course in Figure 6. *Pmar1 *directly inhibits expression of *HesC *(see Figure 1 and Additional file 6), hence *HesC *expression is increased under *Pmar1 *KD and decreased under *Pmar1 *MOE conditions. Although not directly regulated by *Pmar1*, the expression of *TBr*, *Alx1 *and *Ets1 *are positively correlated with perturbations in *Pmar1 *because they are all inhibited by *HesC*.

**Figure 7:**
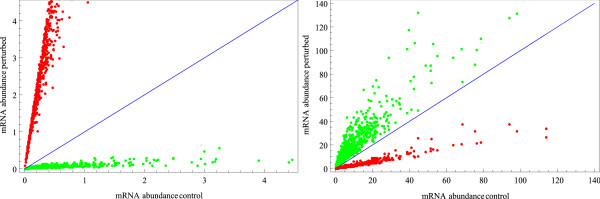
**Scatterplots illustrate the effect of Pmar1-perturbations**. Scatterplots of *HesC *(left) and *Alx1 *(right) mRNA abundance. Simulated abundances at timepoint *t *= 25 *hpf *for all 800 parameter sets are plotted. Red dots represent simulation results under unperturbed (x-axis) and *Pmar1*-KD (y-axis) conditions. Green dots correspond to simulation results under unperturbed (x-axis) and *Pmar1*-MOE (y-axis) conditions. In these examples, the different perturbations are clearly distinguishable. Due to the double-negative regulation (*Pmar1 *inhibits *HesC *inhibits *Alx1*), the perturbations in *Pmar1 *cause converse effects in the expression of *HesC *and *Alx1*.

The same correlation is illustrated in Figure 7, where the abundance under unperturbed and perturbed conditions for one mRNA species at a certain time point is plotted for all parameter sets. The negative correlation between *Pmar1 *and *HesC *expression are shown on the left side, the positive correlation between *Pmar1 *and *Alx1 *expression are shown on the right side. The positive influence of *Pmar1 *expression on the expression of *TBr*, *Alx1 *and *Ets1 *is mediated by the double negative regulation via *HesC*, as described in [25].

**Figure 8:**
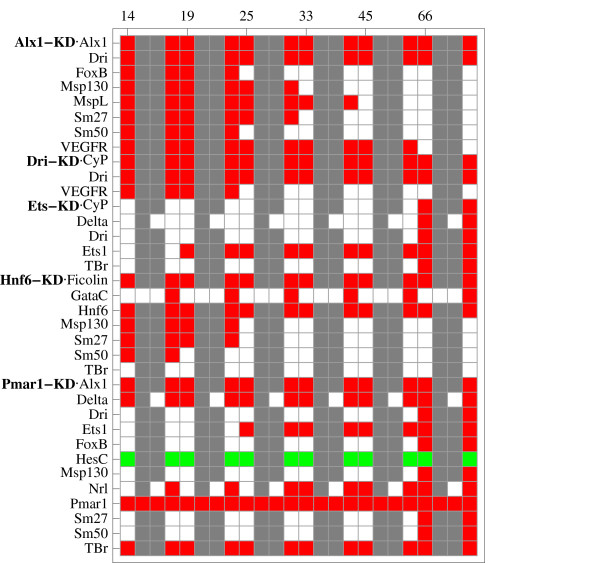
**Territory-specific perturbation effects**. Topological perturbation effects of different genes for *Alx1*-KD, *Dri*-KD, *Ets1*-KD, *Hnf6*-KD and *Pmar1*-KD at different timepoints and for different embryonic territories (all territories, endoderm, mesoderm, PMC). Rows show the effects of the indicated perturbation on the expression of the respective gene at time points 14, 19, 25, 33, 45 and 66 *hpf*. For each timepoint, the 4 columns indicate the effects for (from left to right) 1.) the combination of endoderm, mesoderm and PMC, 2.) endoderm alone, 3.) mesoderm alone, 4.) PMC alone. Red indicates a decrease in expression, green an increase in expression, white indicates no effect of the perturbation on the respective gene. Gray fields indicate genes that are not expressed in the given territory.

**Figure 9:**
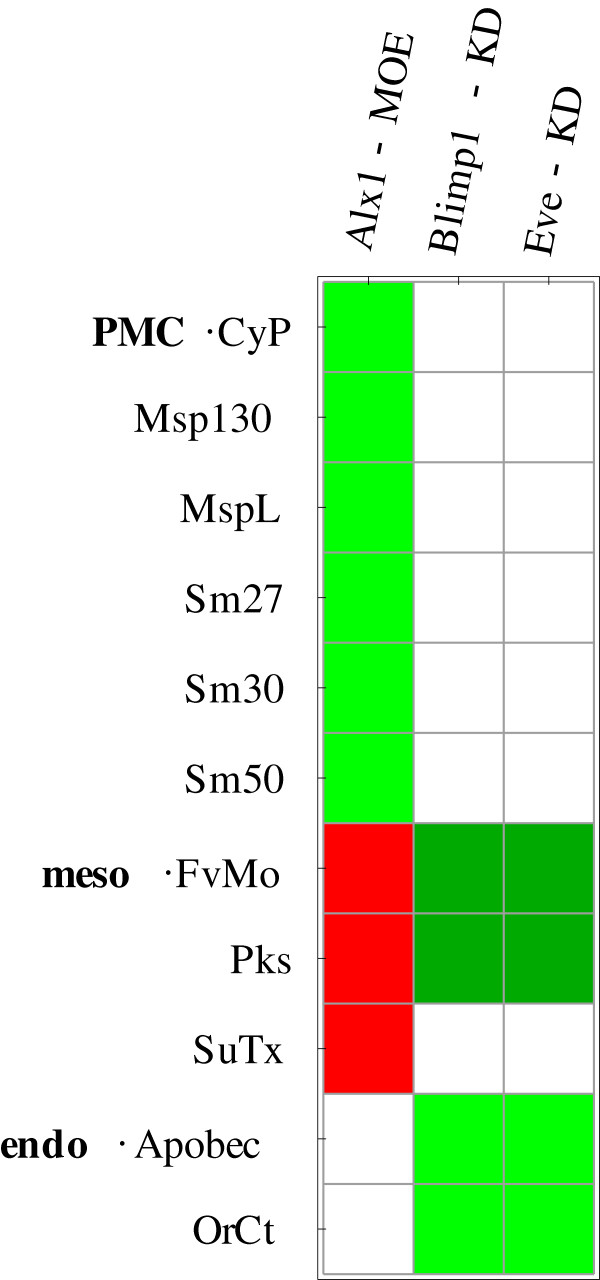
**TPEs discriminate territory-specific downstream differentiation genes**. Groups of genes driven by the Endomesoderm GRN (genes from the lower part of Figure 1) are differently effected by upstream perturbations in simulations. These effects are consistent with the embryonic territory these genes are associated with. The top 6 genes are associated with PMC, the next 3 with mesoderm and the 2 bottom genes with endoderm. TPEs for different timepoints were collated. Red indicates decrease in expression, green indicates increase in expression, dark green indicates a late increase in expression, white indicates no change in expression in response to perturbation.

Visualizations of all simulated perturbation experiments are given in Additional File 2. The results can either be sorted by perturbation, indicating targets of the perturbed gene, or by affected genes, indicating the regulators of a gene. Sorting by perturbation clearly identifies groups of co-regulated genes that are indeed associated with different embryonic territories. The knockdowns of *Alx1*, *Dri*, *Ets1*, *Hnf6 *and *Pmar1 *for example have detectable effects under PMC conditions only (see Figure 8). Genes with coinciding patterns of regulatory effects indicate groups of genes associated with similar territories/regulators. The genes in the downstream differentiation gene batteries (the genes at the bottom of Figure 1 and Additional file 6) can be grouped according to their reaction to different perturbations, as indicated in Figure 9.

**Figure 10:**
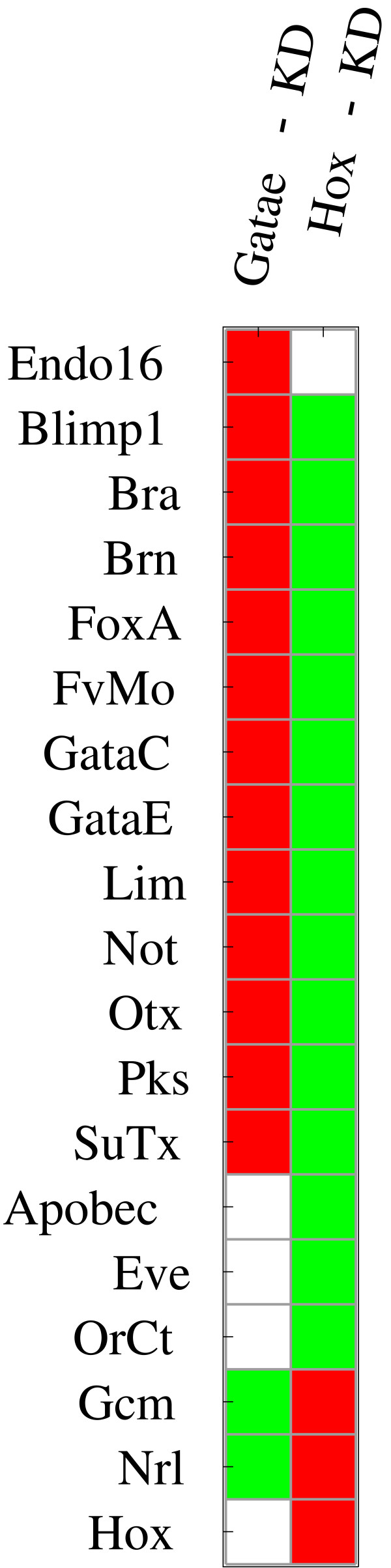
**Negative regulation of GataE by Hox increases amount of TPEs of Hox perturbations**. Topological perturbation effects of *GataE *and *Hox *knockdown on expression of different genes as detected in simulations. Green indicates an increase in expression, red indicates a decrease in expression, white indicates no change in expression. Notice that most genes, including *GataE *are effected by both *GataE*-and *Hox*-KD but that *Hox *expression is not effected by *GataE*-KD.

*GataE *and *Hox *generally have opposite effects, as depicted in Figure 10. This can be explained by the inhibitory role *Hox *has on *GataE *expression. Comparing the vast effects of *Hox*-KD with its relatively limited role in the network topology, it is obvious that a large number of the detected effects are due to the inhibition of *GataE *expression. Such effects are examples for the difficulties that arise in the analysis of large GRNs [6]. In the case of similar ambiguities in experimental data, simulation of additional perturbations can be used to screen for experiments that, according to the current network topology, are most efficient in resolving these.

### Comparison of Experimental and Simulated Perturbation Effects

The qualitative comparison of perturbations either detected experimentally or in simulation using different sampled parameter sets (step 5 in Figure 2) show that the model does not reproduce all experimentally detected effects. The quantitative experimental data were converted to qualitative results using sensible thresholds in order to compare them to the qualitative simulation results (step 6 in Figure 2; see section Methods).

The effects of KD (and overexpression perturbations) in the simulation results are recorded for each of the three territories (endoderm, mesoderm and PMC). As the experimental data only show effects on the whole embryo and not only on a specific territory, we consider as a match the accordance between experimental data and simulated effects in at least one of the territories. This seems reasonable because we omitted the dynamics of intercellular signaling, which might lead to propagation of a perturbation from one territory to another and several genes are only expressed in one territory, both in model and experimental data. Using this scheme to compare simulation and experimental data, we find that 48% of the KD experimental data (43% including MOE) are qualitatively reproduced in our model. We note that the few MOE experiments available compare significantly inferior (only 28% matches with experimental data) to KD experiments (with the exception of *Pmar1 *MOEs), reflecting the unphysiological conditions that are created by injection of mRNA in the embryos, where often amounts are injected that can be several to hundreds of orders of magnitudes higher than the endogenous amounts produced.

**Figure 11:**
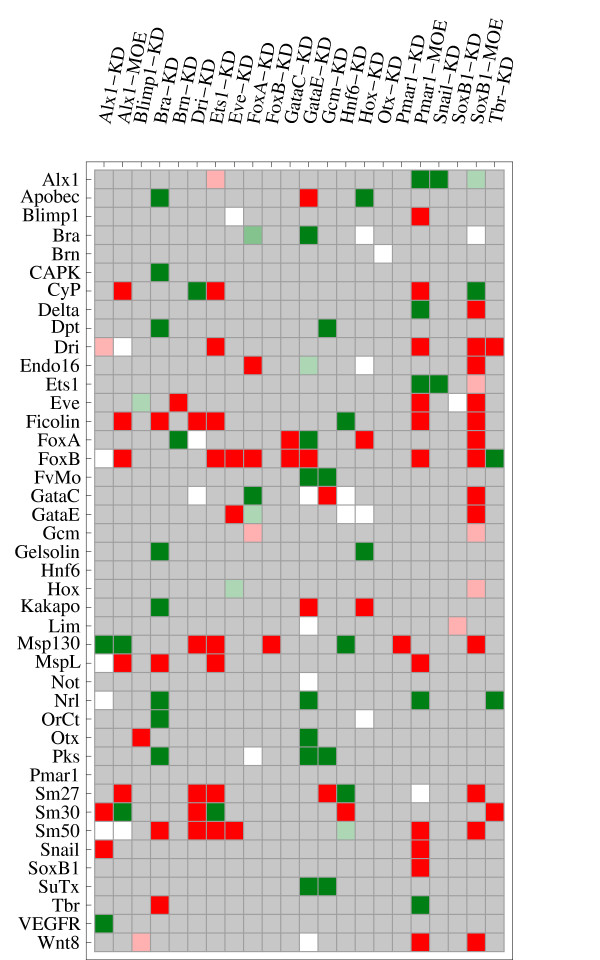
**Detailed comparison of TPEs and experimental data**. Matches and mismatches between experimental data and TPEs. The matches and mismatches for the different timepoints were combined by assigning +1 to matches,-1 to mismatches and calculating the mean for each perturbation/gene pair. Means close to 1 are green, those close to -1 are colored red. Means close to 0 (equal matches/mismatches) are white. Gray cells indicate perturbation/gene pairs for which no experimental data was available. Expression changes for the perturbed gene were not included in the analysis, because translation was blocked in experiments and transcription was blocked in simulations. For updated experimental data, see [24].

A summary of the comparison results is given in Table 1, a visualization is given in Figure 11 and a detailed qualitative overview in Additional File 3. The effects of some perturbations are reproduced very well (*GataE*-KD, *Alx1*-KD and *Bra*-KD), while the effects of other perturbations are reproduced only poorly, worst were the effects of *Ets1*-KD and *Soxb1*-MOE. These two genes are mainly regulated by unknown genes (compare Figure 1 and Additional file 6, inputs labeled 'ubiq'). The regulatory inputs to these genes are not well characterized experimentally and thus demand refinement.

**Table 1:** Summarized comparison of TPEs and experimental data

Perturbation	A	B
Hnf6_KD	9/14	0.6429
Otx_KD	1/2	0.5000
FoxA_KD	9/16	0.5625
TBr_KD	4/7	0.5714
Alx1_KD	11/17	0.6471
Gcm_KD	7/11	0.6364
Hox_KD	6/13	0.4615
Bra_KD	8/12	0.6667
Dri_KD	4/16	0.2500
SoxB1_KD	2/5	0.4000
Ets1_KD	2/18	0.1111
Blimp1_KD	3/9	0.3333
Eve_KD	3/8	0.3750
GataE_KD	19/28	0.6786
Snail_KD	2/2	1.0000
FoxB_KD	0/2	0.0000
GataC_KD	0/2	0.0000
Pmar1_KD	0/2	0.0000
Brn_KD	0/2	0.0000

Pmar1_MOE	11/28	0.3929
Alx1_MOE	5/15	0.3333
SoxB1_MOE	7/39	0.1795

total	113/268	0.4216
total KD	90/186	0.4839
total MOE	23/82	0.2805

Agreement between experimental data and topological perturbation effects. Column A lists matches and total experimental data points, column B indicates the percentage of matches. The last three lines indicate results for all perturbations, for all knockdown perturbations and for all MOE perturbations.

The comparison also indicates genes that frequently react to simulated perturbations as described in experimental data (*Pks*, *Nrl*, *FvMo*, *Alx1 *and *Bra*) and genes for which simulation and experimental data rarely match (*Sm50*, *Sm27 *and *Ficolin*). This behavior might be caused by the large number of upstream interactions varying in strength with the different parameter sets. Other genes, which are important regulatory genes, like *FoxB*, *FoxA *and *Eve *also frequently fail to reproduce the experimental data. These genes have important regulatory roles. Therefore a major conclusion of this analysis is that the wiring of these genes deserves refinement or that the correct regulation here crucially depends on the chosen kinetic parameters.

A comparison of the agreement of simulation results with experimental data for each of the different territories with experimental data reveals that the PMC territory has the highest accordance to experimental data (endoderm: 22.3%, mesoderm: 25%, PMC: 36.5%). This high accordance is most likely due to the extent to which the regulatory interactions in each territory have been investigated. Indeed, the PMC GRN is the best studied part of the network and a recent publication by Oliveri et al. describes a complete PMC GRN [26], which we are currently evaluating on its own.

We need to note that the method presented here is a heuristic approach. Although our results indicate that the Endomesoderm Network topology is not sufficient to reproduce all experimental data, we cannot exclude the possibility that there might exist a choice of kinetic formulas and corresponding parameter values that enables the given topology to perfectly reproduce the experimental data.

In summary, these results indicate the need for a detailed analysis of the regulatory interactions involving *Ets1 *and *SoxB1*, while the regulation of other genes should be investigated in more detail as well (*FoxB*, *FoxA *and *Eve*). Overall, the model reproduces 48% of the experimental data (excluding MOE).

Refinement of model and topology heavily relies on more experimental data. We have simulated the effects of 22 perturbation experiments on the expression of 43 genes. Effects were recorded for 6 time points, yielding 5676 possible perturbation effects from simulation. Of these 5676 possible effects, we could only associate 265 with experimental data. Especially for *Ets1*, *FoxA *and *Eve*, perturbation experiments can be designed for which our model predicts significant effects, listed in Table 2. Some of these effects are listed on [24] under "Genes not affected or shown to be affected only indirectly". Our analysis is not limited to direct effects, so that a comprehensive analysis of the network topology needs to include this data. Such experiments should yield results useful for the improvement of the ODE model and the Endomesoderm Network as well. Besides new experimental data, a careful analysis of the current, detailed experimental data for each interaction in the network and its incorporation into the network is necessary to produce a realistic and quantitative model.

**Table 2:** Predictions for further validation of network topology

perturbation	predicted effect on
*Ets1-KD*	*Delta, TBr*
*FoxA-KD*	*Dpt, FvMo, SuTx*
*Eve*-KD	*FvMo*, *SuTx*, *Apobec*, *Brn*, *Not*, *Nrl*, *OrCT*, *Pks*
*Pmar1-KD*	*Ets1*

Selected detected topological perturbation effects that could not be associated with experimental data. These TPEs can be used as predictions for validation of model and topology with new experimental data.

### Comparison with Updated Network Topology

As the endomesoderm GRN is work in progress, new data and interpretation thereof is constantly added. An updated version of the endomesoderm GRN can be accessed at [4]. The topology underlying our investigation has been published on the web in December 2007. We compared the wiring of the genes that we described as deserving refinement (see above) with their wiring in the actual topology at [4].

We could not find changes in the wiring of *Ets1*, *SoxB1 *and *FoxB*. The wiring of the downstream genes noted above as reproducing experimental data poorly (*Sm50*, *Sm27 *and *Ficolin*) were not changed as well. We did, however find changes in the wiring of *FoxA *(regulatory input by *Hox *instead of *Tgif*) and *Eve *(regulatory input of *Blimp1 *removed). These and other changes in the topology will affect TPEs and their consistence with experimental data. But not all of our concerns have been addressed, like *Ets1 *and *SoxB1*, for example.

Certainly, the updated wiring needs to be addressed in future models that build on the scaffold proposed here. It is, however, out of the scope of this analysis to keep the model up to date with the newest topology at [4]. Our aim is to propose a provisional model that can serve as a scaffold for refining and updating the network and provide a benchmark for judging the topology.

### Comparison with Topologically Randomized Networks

In order to evaluate and rank the agreement between simulation and experimental data, we constructed randomized networks from the Endomesoderm topology and computed their accordance with experimental data. The agreement between the randomized networks tested here and the experimental data is no higher than 23.5%, roughly half as good as between the correct ODE model and experimental data (48%). Two random networks with comparable features were constructed by randomly swapping edges in the original ODE model as described in section Methods and [27] (step 'R' in Figure 2). The randomized networks were subjected to the same method as the original network: conversion to ODE model, simulation with random parameter sets, detection of TPEs, comparison with experimental data. For the original model, using only as little as 50 parameter sets for simulation resulted in a similar agreement with experimental data as when using 800 parameter sets (data not shown). Hence, 100 parameter sets were used for the randomized models to decrease computation time. The randomized models also contained three identical submodels, which only differ in their temporal inputs. The number of edges switched in the randomization was set to 50 times the number of genes in the network, resulting in about 3, 000 exchanges of edges. The randomized models analyzed here were able to reproduce only 20.15% and 23.5% of the experimental data. We also investigated an additional randomized model in which we switched 30, 000 instead of 3, 000 edges and found similar results (data not shown).

Although this agreement with experimental data is in the range of the endoderm part of the original model, no randomized model exceeded this accordance significantly, as does the PMC part of the complete model (see Figure 9). Also, a combination of any three of the randomized networks did not yield an overall accordance with experimental data greater than 25%. We therefore assume that the computed agreement with experimental data of the randomized networks is dependent only on the general topological features of the model (like size and connectivity), the experimental data and the temporal inputs. This indicates that the agreement with experimental data between a model of comparable general features as the one described here with the specified temporal inputs and the experimental data used here can be expected to be less than 25% by chance alone. The accordance with experimental data expected by chance is thus about half the accordance observed using the original topology, indicating significantly improved validity of the Endomesoderm Network compared to random networks.

### Applicability of the Method

The method applied here is generally applicable to any GRN. It is applicable without any prior knowledge of parameters, although a reasonable sampling distribution should be specified.

Using only very limited information, our method is able to extract topological features of a GRN, which can be compared to experimental data. Predictions made by the model can be checked experimentally to refine both ODE model and GRN topology.

In contrast to other, i.e. discrete modeling frameworks, the resulting model can be used for detailed revision and refinement. The refined model can be subjected to the same method for evaluation again, providing a measurement for the resulting increase or decrease in accordance with experimental data. Furthermore, once a satisfying accordance is reached, the constructed network can be used to iteratively estimate parameters of subnetworks. The goal of this improvement must be a fully parameterized ODE model that can be subjected to extended analysis methods. Although the method would require extensive computation when a large number of parameter sets is considered, our analysis shows that only a small fraction of the parameter space needs to be sampled to produce reliable results. We therefore propose to iteratively compute simulation runs and evaluate these until the results converge.

The method is applicable to rather large models for which parameter estimation is not feasible or to models in which different scenarios in the form of different topologies are to be evaluated.

In the case that no comparable models are available, the randomization of the model provides reasonable means of comparison. The computation of robustness to random parameter variations for all genes in the network is a by-product of the computational processes applied here (see Additional File 4). Given a considerable network size and a reasonable limit on parameter values, this robustness might help to identify further features of the network architecture, as described in [28].

### Outlook

As mentioned earlier, the constructed model is by no means sufficient to quantitatively assess the temporal dynamics of the developing sea urchin embryo. In order to improve the model, intercellular effects need to be incorporated in a dynamical way, kinetics for transcriptional activation for each gene must be accurate and parameters for these kinetics need to be determined. We argue that due to the size of the network, improvements should be added in an iterative way, improving the reliability of subnetworks that can be plugged back onto the scaffold.

This can be done by choosing a subnetwork for which extensive experimental data are available (e.g. the PMC part [26]) and parameter estimation is feasible. The subnetwork can be examined in isolation and an improved version can be integrated into the scaffold model. Instead of randomly sampling all parameters, the estimated parameters values can be used and the number of sampled parameters is decreased, increasing the reliability of the analysis. Generally, transcription kinetics can be modified to express the detailed cis-regulatory logic including TF cooperativity or competitive binding. The probability distributions from which parameter values are sampled can be modified to restrict the values of certain parameters to smaller ranges if experimental data suggests this. Using these three approaches, the model can be refined and each iteration of the refinement can be evaluated by comparison with a previous version of the model.

Various approaches for modeling transcriptional activation (see [29] for an example) exist. Most of these general approaches lack details concerning possible cooperativity of TFs, for example by changing the availability of binding sites [30]. We therefore doubt that exclusive use of generalized transcription kinetics as described here and in [29]) are sufficient for a realistic, quantitative model of the Endomesoderm Network.

Once intracellular processes can be modeled satisfactory, the model has to be extended to ensembles of cellular models interacting via extracellular signaling factors. Examples for modeling ensembles of ODE models are given in [19,20].

## Conclusion

If we do understand a biological system, we can create a mathematical model that can reproduce experimental data. If the mathematical model fails to reproduce experimental data, we obviously do not understand all essential parts of the system. We have created a provisional mathematical model of the entire endomesoderm GRN, the first to our knowledge, and used our heuristic to test our understanding of this system. This test results in 48% agreement between simulation results and experimental data. For a correct model, we expect 73.8% agreement with our heurisitc (48% relative to 73.8% is 65%); randomized versions of the model reproduce only 23.5% of the experimental data. We thus conclude that the presented model is only partially correct and that some crucial interactions are not included.

To improve the model, we need to use more realistic transcription kinetics than the stereotypic ones used here. However, the underlying data is not sufficient to unambiguously assign realistic kinetics to all genes in the network. We hope that our provisional model can be used to assign improved kinetics stepwise, starting with genes for which the sufficient data is available (e.g. *Endo16 *[22]). In addition, it is clear that the regulatory interactions in the endomesoderm network are not complete. This is obvious for the genes that still contain inputs termed 'ubiquitous' (e.g. *SoxB1 *or *Ets1*), even in the updated endomesoderm GRN. Consequently, new experimental data is necessary to improve our understanding of gene regulation in the sea urchin embryo. For well established parts of the network (e.g. PMC), detailed data is required. For important regulatory genes like *SoxB1 *and *Ets1*, the missing regulators need to be determined. Model and heuristic proposed here can significantly improve the integration of new data by testing the resulting change in TPEs.

The method we described enables a heuristic assessment of the quality of a network topology generated from perturbation experiments. It allows screening of different topologies due to its low demand in experimental data, before further steps (e.g. parameter estimation) are undertaken. Besides highlighting which parts of the topology agree well with existing experimental data, the model also provides predictions that can aid in the design of new experiments.

Finally, we propose that our method can be used to assess the completeness grade of any network. This could be especially useful for GRNs involved in human diseases, where the amount of connectivity is only unknown and/or many genes/interactions are missing.

## Methods

### Kinetics of the Endomesoderm Network Model

The topology of the Endomesoderm Network was first expressed using Boolean notation. In a second step, this notation is used for the construction of ODE models. The regulatory interactions controlling expression of one gene were integrated based on the Endomesoderm Network as displayed on [4]. We show a simple example for an arbitrary gene: Consider a gene G with two activatory inputs, *A*_1 _and *A*_2 _and one inhibitory input, *I*_1_. If any of the positive inputs is sufficient to activate expression (*A*_1 _∨ *A*_2_) and the activity of the inhibitory input is sufficient to inhibit expression of the target gene, the Boolean expression for the activity of *G *reads

(4)

For the ODE model, we used kinetics as simple as possible. Degradation and translation as well as complex association/dissociation processes were modeled using mass-action kinetics of the form

(5)

We set *k*_*deg *_= 0.3/*hpf *(hours post fertilization), and *k*_*trans *_= 2/*hpf*. The values for complex association/dissociation were sampled along with the parameters regulating transcription.

To translate the formulation of the Boolean model into rate laws, we chose a more sophisticated approach, derived from [31,32]. We use the following elementary modules:

(6)

for activatory inputs *A *on a gene *G *and

(7)

for inhibitory inputs *I*. The constants *c*_*X *_and *k*_*X *_represent individual features of the regulatory role of each gene and TF combination, where *k*_*X *_corresponds to the maximal expression in case of presence of the activator or absence of the inhibitor, respectively. *c*_*X *_is a scaling factor determining the amount of TF necessary to generate a significant change in activity. The elementary modules can be combined to formulate complex regulatory interactions. This is done using multiplication (corresponding to Boolean AND) or addition (corresponding to Boolean OR). The example mentioned above with two activators and one inhibitor thus reads:

(8)

For an extensive list of the transcription kinetics used here, please refer to Additional File 1. Intercellular signaling was modeled as static external inputs defined for each territory. The ODE model uses events to turn external inputs on and off. Instead of changing a concentration directly, we used activatory and inhibitory Hill kinetics for the description of the external inputs. For formal reasons, these Hill kinetics do not depend on some activator or inhibitor but on the simulation time. The change in concentration of an external input is given by

(9)

where *kdeg*·[*x*] is a degradation term. since we require *S*_1 _∈ (0, 1), only one of the two other terms is not equal 0. Thus, by changing Θ and *S*_1_, an external input is activated at time point Θ (in the case that *S*_1 _= 1) or inactivated (when *S*_1 _= 0). By changing *S*_1 _and Θ using events, we can exactly control the activity of the external inputs. The exact numerical values for each of the inputs are given in the SBML Model provided as Additional File 5.

For this analysis, the ODE model was simulated using the simulation tool PyBioS [33,34].

### Detection of Topological Perturbation Effects in ODE Models

TPEs of a model are detected by comparison of different simulation results. To this end, we start by simulating the basic (unperturbed) model. Then we simulate a model perturbed in the expression of one gene, by either KD or MOE, and compare the results. Given an initial ODE model, *M*_0_, the mRNA concentration of a specific mRNA *k *is defined as *s*(*k*, 0, *t*). Knockdown models are created by setting the rate law for the mRNA production in question, *v*_*transcription*_(*t*) to *v*_*transcription*_(*t*)·0.05; over expression is realized by setting *v*_*transcription*_(*t*) to *v*_*transcription*_(*t*) + 2. For a set of *n *perturbations, we get models *M*_*i*_, *i *= 0...*n*, where *M*_0 _denotes the initial, unperturbed model.

A species concentration depends on the initial conditions and parameter sets used. Since the analysis will use multiple parameter sets, we will use *s*(*k, i, j, t*) to denote the concentration of species *k *at time point *t *using model *i *and parameter set *j*.

Let

(10)

where

(11)

By choosing a threshold *th*, we can discriminate between significant and insignificant changes by requiring that  for an effect to be significant.

As stated before, *s*(*k, i, j, t*) depends on the chosen set of parameters. Consequently, *r*(*k, i, j, t*) also depends on the chosen parameter values. In order to find perturbation effects of the model invariant to parameter changes we need to find *r*(*k, i, j, t*) that are significant for arbitrary parameter sets.

Let

(12)

and

(13)

and

(14)

For a topological perturbation effect (TPE) of perturbation *i *on gene *k *at timepoint *t*, we require

(15)

where *ht *is a predefined threshold. If none of the conditions in Equation 15 evaluates to true, no topological perturbation effect is defined for the given combination of *k*, *i*, *t *under parameter set *J*.

### Comparison of Topological Perturbation Effects in the Endomesoderm ODE Model to Experimental Data

We used the algorithm described above to detect TPEs of the ODE model constructed from the Endomesoderm Network. We sampled 800 parameter sets from a lognormal distribution with *σ *= 1.5, *μ *= 0.5. This distribution was in part chosen to avoid too extreme parameter values that could impede the numerical stability of the ODE system. In this study, we set the thresholds *th*_1 _= 1, *th*_2 _= 1.25 and *ht *= 0.9. As an example, the effect of *Pmar1*-KD and -MOE on the expression of *HesC *and *Alx1 *is shown in a scatter plot in Figure 7 for visual orientation.

In order to compare the detected effects to experimental data, we computed the TPEs for a set of time points *T *= (14, 19, 25, 33, 45, 66) reflecting the time intervals used under experimental conditions [24]. TPEs have been computed for the endoderm, mesoderm and PMC parts of the model as well as the total model. These TPEs differ because of the different initial conditions used and the different external inputs. The TPEs detected are of qualitative nature. Expression of a gene is unaffected, increased or decreased by a perturbation. The experimental data is quantitative and often ambiguous. Multiple measurements for one gene, perturbation and time interval are listed. In some cases, up- as well as downregulation was detected at one data point. In order to compare the two sets, we discretized the experimental data. We chose to use only unambiguous data, i.e. data points that were affected in the same way (upregulated, downregulated or unaffected) in all measurements.

The experimental data were determined for the whole embryo. Simulation results were computed for cells of different territories. Combination of these territories to a total simulation result is impossible due to the omitted intercellular signaling. Even a combination of the three different territories could deviate from the experimental result because the embryo is made up of more different cells than just endoderm, mesoderm and PMC. To account for these discrepancies between experimental results and simulation in the comparison of the two sets, we define a match between detected TPEs and experimental results as a TPE detected in at least one of the simulated territories that is equal to the experimentally determined effect.

### Randomization of Network Topology

To generate randomized versions of the model, we applied a method similar to the switching algorithm used in Milo et al. [27] except for not excluding self-edges since the Endomesoderm network itself already contains self-edges. We randomized the ODE model using the mathematical model provided by PyBioS [33,34]. The method chooses two nodes in the network, say genes *g*_*i *_and *g*_*j*_, at random. A regulatory interaction *r*(*x*, *y*) in the network is defined by its origin *x *and target *y*. For *g*_*i *_and *g*_*j*_, two edges, say *r*_1_(*x*, *i*) and *r*_2_(*y*, *j*), that target *g*_*i *_and *g*_*j*_, respectively, are selected at random. The two targets are switched so that *r*_1_(*x*, *i*) → (*x*, *j*) and *r*_2_(*y*, *j*) → (*y*, *i*). This switch is repeated a number of times, usually about 100·*N*, where *N *is the number of nodes in the network [27]. For the Endomesoderm ODE model, each switch is realized consistently in each territory. Note that the borders between the three territories and the external inputs are unaffected by this randomization.

Applying this randomization algorithm, general topological features of the network like the number of nodes and edges, the average node degree and the degree distribution are preserved while the individual wirings are changed. After a randomized network has been constructed, TPEs can be computed and compared with experimental data. In this analysis, we constructed 2 randomized models using 3000 randomization steps and simulated each with 100 different parameter sets. To infer the effect of stronger randomization, we also constructed one model using 30000 randomization steps and simulated it with 20 parameter sets.

## Availability and requirements

The model can be downloaded from the PyBioS website . An SBML version of the model was submitted to the Biomodels database (MODEL2133240427).

## Authors' contributions

All authors contributed to the design and coordination of the study. CK performed the computational implementations and prepared the original draft, which was revised by AJP, EK and CW. AK performed experiments. All authors read and approved the final manuscript.

## Supplementary Material

Additional file 1 **Differential equations of the model**. Differential equations as used in the model. Click here for file

Additional file 2 **All detected TPEs**. All TPEs for all timepoints and territories. Click here for file

Additional file 3 **Detailed comparison of TPEs and exp. Data**. All matches and mismatches between detected TPEs and exp. data. Genes in the network for which no unambiguous expression change due to perturbations could be established from data as of December 2007 are not included. Click here for file

Additional file 4 **Robustness to parameter variations**. Method used to compute the robustness of gene expression against random parameter variations, results and discussion thereof. Click here for file

Additional file 5 **Events used to initiate differential expression**. Listing the events used in setting static inputs to initiate differential expression. For the Hill-Kinetics controlling inputs to the network in each territory (formula 9), we set *S*_1 _= 1, *S*_2 _= 0 at the time given in the respective 'on'-column and we set *S*_1 _= 0, *S*_2 _= 1 at the time given in the respective 'off'-column. Empty cells indicate inputs that are off (*S*_1 _= 0, *S*_2 _= 1) for the whole simulation time. Click here for file

Additional file 6 **SBML Model**. Model in SBML format. Parameter values are set to arbitrary values. Also available on BioModels under ID MODEL2133240427. Click here for file
