# Supplementary material for: Monte Carlo analysis of an ODE Model of the Sea Urchin Endomesoderm Network
Source: BMC Syst Biol. 2009 Aug 23;3:83. doi: 10.1186/1752-0509-3-83 (PMC2739852; doi:10.1186/1752-0509-3-83)
Supplement: Additional file 1 — Differential equations of the model. Differential equations as used in the model. [file 1752-0509-3-83-S1.pdf]

Monte Carlo analysis of an ODE Model of the Sea Urchin Endomesoderm Network
Supplementary Table 1: Differential Equations

May 19, 2009

$$v_{Gcad_{INPUT}}(t) = \frac{S1_{Gcad}\cdot HillK_{Gcad}\cdot t^{HillH_{Gcad}}}{\theta_{1Gcad}^{HillH_{Gcad}+_{t}HillHGcad}} + S2_{Gcad}\cdot HillK_{Gcad}\cdot(1-\frac{t^{HillH_{Gcad}}}{theta2Gcad^{HillH_{Gcad}+t^{HillH_{Gcad})}}})$$

$$v_{Notch_{INPUT}}(t) = \frac{S1_{Notch}\cdot HillK_{Notch}\cdot t^{HillH_{Notch}}}{\theta_{1Notch}^{HillH_{Notch}+_{t}HillHNotch}} + S2_{Notch}\cdot HillK_{Notch}\cdot(1-\frac{t^{HillH_{Notch}}}{theta2Notch^{HillH_{Notch}+t^{HillH_{Notch})}}})$$

$$v_{Otx_{INPUT}}(t) = \frac{S1_{Otx}\cdot HillK_{Otx}\cdot t^{HillH_{Otx}}}{\theta_{1Otx}^{HillH_{Otx}+_{t}HillHOtx}} + S2_{Otx}\cdot HillK_{Otx}\cdot(1-\frac{t^{HillH_{Otx}}}{theta2Otx^{HillH_{Otx}+t^{HillH_{Otx})}}})$$

$$v_{SoxB1_{INPUT}}(t) = \frac{S1_{SoxB1}\cdot HillK_{SoxB1}\cdot t^{HillH_{SoxB1}}}{\theta_{1SoxB1}^{HillH_{SoxB1}+_{t}HillHSoxB1}} + S2_{SoxB1}\cdot HillK_{SoxB1}\cdot(1-\frac{t^{HillH_{SoxB1}}}{theta2SoxB1^{HillH_{SoxB1}+t^{HillH_{SoxB1})}}})$$

$$v_{SuH_{INPUT}}(t) = \frac{S1_{SuH}\cdot HillK_{SuH}\cdot t^{HillH_{SuH}}}{\theta_{1SuH}^{HillH_{SuH}+_{t}HillHSuH}} + S2_{SuH}\cdot HillK_{SuH}\cdot(1-\frac{t^{HillH_{SuH}}}{theta2SuH^{HillH_{SuH}+t^{HillH_{SuH})}}})$$

$$v_{UMR_{INPUT}}(t) = \frac{S1_{UMR}\cdot HillK_{UMR}\cdot t^{HillH_{UMR}}}{\theta_{1UMR}^{HillH_{UMR}+_{t}HillHUMR}} + S2_{UMR}\cdot HillK_{UMR}\cdot(1-\frac{t^{HillH_{UMR}}}{theta2UMR^{HillH_{UMR}+t^{HillH_{UMR})}}})$$

$$v_{UVAOtx_{INPUT}}(t) = \frac{S1_{UVAOtx}\cdot HillK_{UVAOtx}\cdot t^{HillH_{UVAOtx}}}{\theta_{1UVAOtx}^{HillH_{UVAOtx}+_{t}HillHUVAOtx}} + S2_{UVAOtx}\cdot HillK_{UVAOtx}\cdot(1-\frac{t^{HillH_{UVAOtx}}}{theta2UVAOtx^{HillH_{UVAOtx}+t^{HillH_{UVAOtx})}}})$$

$$v_{UbiqSoxB1_{INPUT}}(t) = \frac{S1_{UbiqSoxB1}\cdot HillK_{UbiqSoxB1}\cdot t^{HillH_{UbiqSoxB1}}}{\theta_{1UbiqSoxB1}^{HillH_{UbiqSoxB1}+_{t}HillHUbiqSoxB1}} + S2_{UbiqSoxB1}\cdot HillK_{UbiqSoxB1}\cdot(1-\frac{t^{HillH_{UbiqSoxB1}}}{theta2UbiqSoxB1^{HillH_{UbiqSoxB1}+t^{HillH_{UbiqSoxB1})}}})$$

$$v_{VEGF_{INPUT}}(t) = \frac{S1_{VEGF}\cdot HillK_{VEGF}\cdot t^{HillH_{VEGF}}}{\theta_{1VEGF}^{HillH_{VEGF}+_{t}HillHVEGF}} + S2_{VEGF}\cdot HillK_{VEGF}\cdot(1-\frac{t^{HillH_{VEGF}}}{theta2VEGF^{HillH_{VEGF}+t^{HillH_{VEGF})}}})$$

$$v_{cB_{INPUT}}(t) = \frac{S1_{cB}\cdot HillK_{cB}\cdot t^{HillH_{cB}}}{\theta_{1cB}^{HillH_{cB}+_{t}HillHcB}} + S2_{cB}\cdot HillK_{cB}\cdot(1-\frac{t^{HillH_{cB}}}{theta2cB^{HillH_{cB}+t^{HillH_{cB})}}})$$

$$v_{Alx1_{transcription}}(t) = (\frac{k_{Ets1_P}\cdot Ets1_p(t)}{c_{Ets1_P}+Ets1_p(t)} + \frac{k_{UbiqAlx1_P}\cdot UbiqAlx1_p(t)}{c_{UbiqAlx1_P}+UbiqAlx1_p(t)} + \frac{k_{Tgif_P}\cdot Tgif_p(t)}{c_{Tgif_P}+Tgif_p(t)})\cdot\frac{k_{Gcm_P}\cdot c_{Gcm_P}}{c_{Gcm_P}+Gcm_p(t)}\cdot\frac{k_{HesC_P}\cdot c_{HesC_P}}{c_{HesC_P}+HesC_p(t)}$$

$$v_{Apobec_{transcription}}(t)\begin{cases} = \frac{k_{Bra_P}\cdot Bra_p(t)}{c_{Bra_P}+Bra_p(t)}\cdot\frac{k_{Hox_P}\cdot c_{Hox_P}}{c_{Hox_P}+Hox_p(t)}, & \text{in endoderm, PMC}\\ = (\frac{k_{Bra_P}\cdot Bra_p(t)}{c_{Bra_P}+Bra_p(t)} + \frac{k_{Bra_P^{endo}}\cdot Bra_p^{endo}(t)}{c_{Bra_P^{endo}}\cdot Bra_p^{endo}(t)})\cdot\frac{k_{Hox_P}\cdot c_{Hox_P}}{c_{Hox_P}+Hox_p(t)}, & \text{in mesoderm}\end{cases}$$

$$\mathrm{v}_{Blimp1_{transcription}}(t) = (\frac{k_{Otx_P}\cdot Otx_p(t)}{c_{Otx_P}+Otx_p(t)} + \frac{k_{Brn_P}\cdot Brn_p(t)}{c_{Brn_P}+Brn_p(t)} + \frac{k_{GataE_P}\cdot GataE_p(t)}{c_{GataE_P}+GataE_p(t)} + \frac{k_{nBTCF_P}\cdot nBTCF_p(t)}{c_{nBTCF_P}+nBTCF_p(t)} + \frac{k_{Eve_P}\cdot Eve_p(t)}{c_{Eve_P}+Eve_p(t)})\cdot\frac{k_{Blimp1_P}\cdot c_{Blimp1_P}}{c_{Blimp1_P}+Blimp1_p(t)}\cdot\frac{k_{GroTCF_P}\cdot c_{GroTCF_P}}{c_{GroTCF_P}+GroTCF_p(t)}$$

$$v_{Bra_{transcription}}(t) = (\frac{k_{GataE_P}\cdot GataE_p(t)}{c_{GataE_P}+GataE_p(t)} + \frac{k_{nBTCF_P}\cdot nBTCF_p(t)}{c_{nBTCF_P}+nBTCF_p(t)} + \frac{k_{Otx_P}\cdot Otx_p(t)}{c_{Otx_P}+Otx_p(t)})\cdot\frac{k_{GroTCF_P}\cdot c_{GroTCF_P}}{c_{GroTCF_P}+GroTCF_p(t)}$$

$$v_{Brn_{transcription}}(t) = \frac{k_{GataE_P}\cdot GataE_p(t)}{c_{GataE_P}+GataE_p(t)}$$

$$v_{CAPK_{transcription}}(t)\begin{cases} = 0, & \text{in endoderm, PMC}\\ = \frac{k_{Bra_P^{endo}}\cdot Bra_p^{endo}(t)}{c_{Bra_P^{endo}}+Bra_p^{endo}(t)}, & \text{in mesoderm}\end{cases}$$

$$v_{CyP_{transcription}}(t) = (\frac{k_{Dri_P}\cdot Dri_p(t)}{c_{Dri_P}+Dri_p(t)} + \frac{k_{Ets1_P}\cdot Ets1_p(t)}{c_{Ets1_P}+Ets1_p(t)})\cdot\frac{k_{SoxB1_P}\cdot c_{SoxB1_P}}{c_{SoxB1_P}+SoxB1_p(t)}$$

$$v_{Delta_{transcription}}(t) = (\frac{k_{UbiqDelta_P}\cdot UbiqDelta_p(t)}{c_{UbiqDelta_P}+UbiqDelta_p(t)} + \frac{k_{UMADelta_P}\cdot UMADelta_p(t)}{c_{UMADelta_P}+UMADelta_p(t)} + \frac{k_{Ets1_P}\cdot Ets1_p(t)}{c_{Ets1_P}+Ets1_p(t)})\cdot\frac{k_{HesC_P}\cdot c_{HesC_P}}{c_{HesC_P}+HesC_p(t)}$$

$$v_{Dpt_{transcription}}(t)\begin{cases} = \frac{k_{Gcm_P}\cdot Gcm_p(t)}{c_{Gcm_P}+Gcm_p(t)}, & \text{in endoderm, PMC}\\ = \frac{k_{Bra_P^{endo}}\cdot Bra_p^{endo}(t)}{c_{Bra_P^{endo}}+Bra_p^{endo}(t)} + \frac{k_{Gcm_P}\cdot Gcm_p(t)}{c_{Gcm_P}+Gcm_p(t)}, & \text{in mesoderm}\end{cases}$$

$$v_{Dri_{transcription}}(t) = \frac{k_{Alx1_P}\cdot Alx1_p(t)}{c_{Alx1_P}+Alx1_p(t)} + \frac{k_{Ets1_P}\cdot Ets1_p(t)}{c_{Ets1_P}+Ets1_p(t)}$$

$$v_{ES_{transcription}}(t) = (\frac{k_{Dri_P}\cdot Dri_p(t)}{c_{Dri_P}+Dri_p(t)} + \frac{k_{UbiqES_P}\cdot UbiqES_p(t)}{c_{UbiqES_P}+UbiqES_p(t)})\cdot\frac{k_{HesC_P}\cdot c_{HesC_P}}{c_{HesC_P}+HesC_p(t)}$$

$$v_{Endo16_{transcription}}(t) = \frac{k_{Otx_P}\cdot Otx_p(t)}{c_{Otx_P}+Otx_p(t)} + \frac{k_{Brn_P}\cdot Brn_p(t)}{c_{Brn_P}+Brn_p(t)}$$

$$v_{Erg_{transcription}}(t) = \frac{k_{TBr_P}\cdot TBr_p(t)}{c_{TBr_P}+TBr_p(t)} + \frac{k_{Ets1_P}\cdot Ets1_p(t)}{c_{Ets1_P}+Ets1_p(t)} + \frac{k_{Hex_P}\cdot Hex_p(t)}{c_{Hex_P}+Hex_p(t)}$$

$$v_{Ets1_{transcription}}(t) = \frac{k_{UbiqEts1_P} \cdot UbiqEts1_p(t)}{c_{UbiqEts1_P} + UbiqEts1_p(t)} \cdot \frac{k_{HesC_P} \cdot c_{HesC_P}}{c_{HesC_P} + HesC_p(t)}$$

$$v_{Eve_{transcription}}(t) = (\frac{k_{Blimp1_P} \cdot Blimp1_p(t)}{c_{Blimp1_P} + Blimp1_p(t)} + \frac{k_{nBTCF_P} \cdot nBTCF_p(t)}{c_{nBTCF_P} + nBTCF_p(t)}) \cdot \frac{k_{GroTCF_P} \cdot c_{GroTCF_P}}{c_{GroTCF_P} + GroTCF_p(t)} \cdot \frac{k_{Hox_P} \cdot c_{Hox_P}}{c_{Hox_P} + Hox_p(t)}$$

$$v_{Ficolin_{transcription}}(t) = \frac{k_{Ets1_P} \cdot Ets1_p(t)}{c_{Ets1_P} + Ets1_p(t)} + \frac{k_{Hnf6_P} \cdot Hnf6_p(t)}{c_{Hnf6_P} + Hnf6_p(t)} + \frac{k_{Hex_P} \cdot Hex_p(t)}{c_{Hex_P} + Hex_p(t)} + \frac{k_{Erg_P} \cdot Erg_p(t)}{c_{Erg_P} + Erg_p(t)}$$

$$v_{FoxA_{transcription}}(t) = (\frac{k_{GataE_P} \cdot GataE_p(t)}{c_{GataE_P} + GataE_p(t)} + \frac{k_{nBTCF_P} \cdot nBTCF_p(t)}{c_{nBTCF_P} + nBTCF_p(t)} + \frac{k_{Otx_P} \cdot Otx_p(t)}{c_{Otx_P} + Otx_p(t)} + \frac{k_{Bra_P} \cdot Bra_p(t)}{c_{Bra_P} + Bra_p(t)} + \frac{k_{Tgif_P} \cdot Tgif_p(t)}{c_{Tgif_P} + Tgif_p(t)}) \cdot \frac{k_{GroTFC_P} \cdot c_{GroTFC_P}}{c_{GroTFC_P} + GroTFC_p(t)} \cdot \frac{k_{FoxA_P} \cdot c_{FoxA_P}}{c_{FoxA_P} + FoxA_p(t)}$$

$$v_{FoxB_{transcription}}(t) = (\frac{k_{Alx1_P} \cdot Alx1_p(t)}{c_{Alx1_P} + Alx1_p(t)} + \frac{k_{Ets1_P} \cdot Ets1_p(t)}{c_{Ets1_P} + Ets1_p(t)} + \frac{k_{TBr_P} \cdot TBr_p(t)}{c_{TBr_P} + TBr_p(t)} + \frac{k_{Dri_P} \cdot Dri_p(t)}{c_{Dri_P} + Dri_p(t)}) \cdot \frac{k_{FoxB_P} \cdot c_{FoxB_P}}{c_{FoxB_P} + FoxB_p(t)}$$

$$v_{FoxN23_{transcription}}(t) = \frac{k_{nBTCF_P} \cdot nBTCF_p(t)}{c_{nBTCF_P} + nBTCF_p(t)}$$

$$v_{FoxO_{transcription}}(t) = \frac{k_{Ets1_P} \cdot Ets1_p(t)}{c_{Ets1_P} + Ets1_p(t)} + \frac{k_{Hex_P} \cdot Hex_p(t)}{c_{Hex_P} + Hex_p(t)} + \frac{k_{Tgif_P} \cdot Tgif_p(t)}{c_{Tgif_P} + Tgif_p(t)} + \frac{k_{Erg_P} \cdot Erg_p(t)}{c_{Erg_P} + Erg_p(t)}$$

$$v_{FvMo_{transcription}}(t) = \frac{k_{Gcm_P} \cdot Gcm_p(t)}{c_{Gcm_P} + Gcm_p(t)} + \frac{k_{GataE_P} \cdot GataE_p(t)}{c_{GataE_P} + GataE_p(t)}$$

$$v_{GataC_{transcription}}(t) = (\frac{k_{GataE_P} \cdot GataE_p(t)}{c_{GataE_P} + GataE_p(t)} + \frac{k_{Hnf6_P} \cdot Hnf6_p(t)}{c_{Hnf6_P} + Hnf6_p(t)} + \frac{k_{GCM_P} \cdot GCM_p(t)}{c_{GCM_P} + GCM_p(t)}) \cdot \frac{k_{GataC_P} \cdot c_{GataC_P}}{c_{GataC_P} + GataC_p(t)}$$

$$v_{GataE_{transcription}}(t) = (\frac{k_{Otx_P} \cdot Otx_p(t)}{c_{Otx_P} + Otx_p(t)} + \frac{k_{SuHN_P} \cdot SuHN_p(t)}{c_{SuHN_P} + SuHN_p(t)}) \cdot \frac{k_{Hox_P} \cdot c_{Hox_P}}{c_{Hox_P} + Hox_p(t)}$$

$$v_{Gcad_{transcription}}(t) = \frac{k_{UbiqGcad_P} \cdot UbiqGcad_p(t)}{c_{UbiqGcad_P} + UbiqGcad_p(t)} \cdot \frac{k_{Snail_P} \cdot c_{Snail_P}}{c_{Snail_P} + Snail_p(t)}$$

$$v_{Gcm_{transcription}}(t) = (\frac{k_{nBTCF_P} \cdot nBTCF_p(t)}{c_{nBTCF_P} + nBTCF_p(t)} + \frac{k_{SuHN_P} \cdot SuHN_p(t)}{c_{SuHN_P} + SuHN_p(t)} + \frac{k_{Gcm_P} \cdot Gcm_p(t)}{c_{Gcm_P} + Gcm_p(t)}) \cdot \frac{k_{GroTCF_P} \cdot c_{GroTCF_P}}{c_{GroTCF_P} + GroTCF_p(t)} \cdot \frac{k_{FoxA_P} \cdot c_{FoxA_P}}{c_{FoxA_P} + FoxA_p(t)} \cdot \frac{k_{Alx1_P} \cdot c_{Alx1_P}}{c_{Alx1_P} + Alx1_p(t)}$$

$$v_{Gelsolin_{transcription}}(t) \begin{cases} = \frac{k_{Bra_P} \cdot Bra_p(t)}{c_{Bra_P} + Bra_p(t)}, & \text{in endoderm, PMC} \\ = \frac{k_{Bra_P} \cdot Bra_p(t)}{c_{Bra_P} + Bra_p(t)} + \frac{k_{Bra_P^{endo}} \cdot Bra_p^{endo}(t)}{c_{Bra_P^{endo}} + Bra_p^{endo}(t)}, & \text{in mesoderm} \end{cases}$$

$$v_{HesC_{transcription}}(t) = \frac{k_{UbiqHesC_P} \cdot UbiqHesC_p(t)}{c_{UbiqHesC_P} + UbiqHesC_p(t)} \cdot \frac{k_{Pmar1_P} \cdot c_{Pmar1_P}}{c_{Pmar1_P} + Pmar1_p(t)}$$

$$v_{Hex_{transcription}}(t) = \frac{k_{Tgif_P} \cdot Tgif_p(t)}{c_{Tgif_P} + Tgif_p(t)} + \frac{k_{Ets1_P} \cdot Ets1_p(t)}{c_{Ets1_P} + Ets1_p(t)} + \frac{k_{Erg_P} \cdot Erg_p(t)}{c_{Erg_P} + Erg_p(t)}$$

$$v_{Hnf6_{transcription}}(t) = \frac{k_{UbiqHnf6_P} \cdot UbiqHnf6_p(t)}{c_{UbiqHnf6_P} + UbiqHnf6_p(t)}$$

$$v_{Hox_{transcription}}(t) = (\frac{k_{Blimp1_P} \cdot Blimp1_p(t)}{c_{Blimp1_P} + Blimp1_p(t)} + \frac{k_{nBTCF_P} \cdot nBTCF_p(t)}{c_{nBTCF_P} + nBTCF_p(t)} + \frac{k_{Eve_P} \cdot Eve_p(t)}{c_{Eve_P} + Eve_p(t)} + \frac{k_{Otx_P} \cdot Otx_p(t)}{c_{Otx_P} + Otx_p(t)}) \cdot \frac{k_{GroTCF_P} \cdot c_{GroTCF_P}}{c_{GroTCF_P} + GroTCF_p(t)}$$

$$v_{Kakapo_{transcription}}(t) \begin{cases} = \frac{k_{Bra_P} \cdot Bra_p(t)}{c_{Bra_P} + Bra_p(t)}, & \text{in endoderm, PMC} \\ = \frac{k_{Bra_P} \cdot Bra_p(t)}{c_{Bra_P} + Bra_p(t)} + \frac{k_{Bra_P^{endo}} \cdot Bra_p^{endo}(t)}{c_{Bra_P^{endo}} + Bra_p^{endo}(t)}, & \text{in mesoderm} \end{cases}$$

$$v_{Lim_{transcription}}(t) = \frac{k_{GataE_P} \cdot GataE_p(t)}{c_{GataE_P} + GataE_p(t)} + \frac{k_{Otx_P} \cdot Otx_p(t)}{c_{Otx_P} + Otx_p(t)}$$

$$v_{Msp130_{transcription}}(t) = \frac{k_{Hnf6_P} \cdot Hnf6_p(t)}{c_{Hnf6_P} + Hnf6_p(t)} + \frac{k_{FoxB_P} \cdot FoxB_p(t)}{c_{FoxB_P} + FoxB_p(t)} + \frac{k_{Ets1_P} \cdot Ets1_p(t)}{c_{Ets1_P} + Ets1_p(t)} + \frac{k_{Alx1_P} \cdot Alx1_p(t)}{c_{Alx1_P} + Alx1_p(t)} + \frac{k_{Hex_P} \cdot Hex_p(t)}{c_{Hex_P} + Hex_p(t)} + \frac{k_{TBr_P} \cdot TBr_p(t)}{c_{TBr_P} + TBr_p(t)} + \frac{k_{Erg_P} \cdot Erg_p(t)}{c_{Erg_P} + Erg_p(t)}$$

$$v_{MspL_{transcription}}(t) = \frac{k_{Ets1_P} \cdot Ets1_p(t)}{c_{Ets1_P} + Ets1_p(t)} + \frac{k_{Alx1_P} \cdot Alx1_p(t)}{c_{Alx1_P} + Alx1_p(t)} + \frac{k_{VEGFSignal_P} \cdot VEGFSignal_p(t)}{c_{VEGFSignal_P} + VEGFSignal_p(t)} + \frac{k_{Hex_P} \cdot Hex_p(t)}{c_{Hex_P} + Hex_p(t)} + \frac{k_{Erg_P} \cdot Erg_p(t)}{c_{Erg_P} + Erg_p(t)}$$

$$v_{Not_{transcription}}(t) = \frac{k_{GataE_P} \cdot GataE_p(t)}{c_{GataE_P} + GataE_p(t)}$$

$$v_{Nrl_{transcription}}(t)\begin{cases} = (\frac{(k_{TBr_P} \cdot TBr_p(t)}{c_{TBr_P}+TBr_p(t)} + \frac{k_{UMANrl_P} \cdot UMANrl_p(t)}{c_{UMANrl_P}+UMANrl_p(t)} + \frac{k_{FoxN23_P} \cdot FoxN23_p(t)}{c_{FoxN23_P}+FoxN23_p(t)}) \cdot \frac{k_{GataE_P} \cdot c_{GataE_P}}{c_{GataE_P}+GataE_p(t)} \cdot \frac{k_{HesC_P} \cdot c_{HesC_P}}{c_{HesC_P}+HesC_p(t)} \cdot \frac{k_{Tgif_P} \cdot c_{Tgif_P}}{c_{Tgif_P}+Tgif_p(t)}, \\ \qquad \text{in endoderm, PMC} \\ = (\frac{(k_{TBr_P} \cdot TBr_p(t)}{c_{TBr_P}+TBr_p(t)} + \frac{k_{UMANrl_P} \cdot UMANrl_p(t)}{c_{UMANrl_P}+UMANrl_p(t)} + \frac{k_{FoxN23_P} \cdot FoxN23_p(t)}{c_{FoxN23_P}+FoxN23_p(t)}) \cdot \frac{k_{GataE_P} \cdot c_{GataE_P}}{c_{GataE_P}+GataE_p(t)} \cdot \frac{k_{HesC_P} \cdot c_{HesC_P}}{c_{HesC_P}+HesC_p(t)} + \frac{k_{Bra^{endo}_P} \cdot Bra^{endo}_p(t)}{c_{Bra^{endo}_P}+Bra^{endo}_p(t)} \cdot \frac{k_{Tgif_P} \cdot c_{Tgif_P}}{c_{Tgif_P}+Tgif_p(t)}, \\ \qquad \text{in mesoderm} \end{cases}$$

$$v_{OrCt_{transcription}}(t)\begin{cases} = \frac{k_{Bra_P} \cdot Bra_p(t)}{c_{Bra_P}+Bra_p(t)} \cdot \frac{k_{Hox_P} \cdot c_{Hox_P}}{c_{Hox_P}+Hox_p(t)}, & \text{in endoderm, PMC} \\ = (\frac{k_{Bra_P} \cdot Bra_p(t)}{c_{Bra_P}+Bra_p(t)} + \frac{k_{Bra^{endo}_P} \cdot Bra^{endo}_p(t)}{c_{Bra^{endo}_P}+Bra^{endo}_p(t)}) \cdot \frac{k_{Hox_P} \cdot c_{Hox_P}}{c_{Hox_P}+Hox_p(t)}, & \text{in mesoderm} \end{cases}$$

$$v_{Otx_{transcription}}(t) = \frac{k_{UVAOtx_P} \cdot UVAOtx_p(t)}{c_{UVAOtx_P}+UVAOtx_p(t)} + \frac{k_{Blimp1_P} \cdot Blimp1_p(t)}{c_{Blimp1_P}+Blimp1_p(t)} + \frac{k_{GataE_P} \cdot GataE_p(t)}{c_{GataE_P}+GataE_p(t)} + \frac{k_{Otx_P} \cdot Otx_p(t)}{c_{Otx_P}+Otx_p(t)}$$

$$v_{Pks_{transcription}}(t)\begin{cases} = \frac{k_{Gcm_P} \cdot Gcm_p(t)}{c_{Gcm_P}+Gcm_p(t)} + \frac{k_{GataE_P} \cdot GataE_p(t)}{c_{GataE_P}+GataE_p(t)}, & \text{in endoderm, PMC} \\ = \frac{k_{Bra^{endo}_P} \cdot Bra^{endo}_p(t)}{c_{Bra^{endo}_P}+Bra^{endo}_p(t)} + \frac{k_{Gcm_P} \cdot Gcm_p(t)}{c_{Gcm_P}+Gcm_p(t)} + \frac{k_{GataE_P} \cdot GataE_p(t)}{c_{GataE_P}+GataE_p(t)}, & \text{in mesoderm} \end{cases}$$

$$v_{Pmar1_{transcription}}(t) = (\frac{k_{nBTCF_P} \cdot nBTCF_p(t)}{c_{nBTCF_P}+nBTCF_p(t)} + \frac{k_{Otx_P} \cdot Otx_p(t)}{c_{Otx_P}+Otx_p(t)}) \cdot \frac{k_{GroTCF_P} \cdot c_{GroTCF_P}}{c_{GroTCF_P}+GroTCF_p(t)}$$

$$v_{Sm27_{transcription}}(t) = \frac{k_{Dri_P} \cdot Dri_p(t)}{c_{Dri_P}+Dri_p(t)} + \frac{k_{Hnf6_P} \cdot Hnf6_p(t)}{c_{Hnf6_P}+Hnf6_p(t)} + \frac{k_{Ets1_P} \cdot Ets1_p(t)}{c_{Ets1_P}+Ets1_p(t)} + \frac{k_{Alx1_P} \cdot Alx1_p(t)}{c_{Alx1_P}+Alx1_p(t)} + \frac{k_{Tel_P} \cdot Tel_p(t)}{c_{Tel_P}+Tel_p(t)} + \frac{k_{Hex_P} \cdot Hex_p(t)}{c_{Hex_P}+Hex_p(t)} + \frac{k_{Erg_P} \cdot Erg_p(t)}{c_{Erg_P}+Erg_p(t)}$$

$$v_{Sm30_{transcription}}(t) = \frac{k_{VEGFSignal_P} \cdot VEGFSignal_p(t)}{c_{VEGFSignal_P}+VEGFSignal_p(t)}$$

$$v_{Sm50_{transcription}}(t) = \frac{k_{Dri_P} \cdot Dri_p(t)}{c_{Dri_P}+Dri_p(t)} + \frac{k_{Hnf6_P} \cdot Hnf6_p(t)}{c_{Hnf6_P}+Hnf6_p(t)} + \frac{k_{Ets1_P} \cdot Ets1_p(t)}{c_{Ets1_P}+Ets1_p(t)} + \frac{k_{Alx1_P} \cdot Alx1_p(t)}{c_{Alx1_P}+Alx1_p(t)} + \frac{k_{Tel_P} \cdot Tel_p(t)}{c_{Tel_P}+Tel_p(t)} + \frac{k_{Hex_P} \cdot Hex_p(t)}{c_{Hex_P}+Hex_p(t)} + \frac{k_{Erg_P} \cdot Erg_p(t)}{c_{Erg_P}+Erg_p(t)} + \frac{k_{VEGFSignal_P} \cdot VEGFSignal_p(t)}{c_{VEGFSignal_P}+VEGFSignal_p(t)}$$

$$v_{Snail_{transcription}}(t) = \frac{k_{Hex_P} \cdot Hex_p(t)}{c_{Hex_P}+Hex_p(t)}$$

$$v_{SoxB1_{transcription}}(t) = \frac{k_{UbiqSoxB1_P} \cdot UbiqSoxB1_p(t)}{c_{UbiqSoxB1_P}+UbiqSoxB1_p(t)} \cdot \frac{k_{SoxB1_P} \cdot c_{SoxB1_P}}{c_{SoxB1_P}+SoxB1_p(t)}$$

$$v_{SoxC_{transcription}}(t) = \frac{k_{UbiqSoxC_P} \cdot UbiqSoxC_p(t)}{c_{UbiqSoxC_P}+UbiqSoxC_p(t)} \cdot \frac{k_{HesC_P} \cdot c_{HesC_P}}{c_{HesC_P}+HesC_p(t)} \cdot \frac{k_{SoxC_P} \cdot c_{SoxC_P}}{c_{SoxC_P}+SoxC_p(t)}$$

$$v_{SuTx_{transcription}}(t) = \frac{k_{Gcm_P} \cdot Gcm_p(t)}{c_{Gcm_P}+Gcm_p(t)} + \frac{k_{GataE_P} \cdot GataE_p(t)}{c_{GataE_P}+GataE_p(t)}$$

$$v_{TBr_{transcription}}(t) = \frac{k_{Ets1_P} \cdot Ets1_p(t)}{c_{Ets1_P}+Ets1_p(t)} \cdot \frac{k_{HesC_P} \cdot c_{HesC_P}}{c_{HesC_P}+HesC_p(t)} \cdot \frac{k_{TBr_P} \cdot c_{TBr_P}}{c_{TBr_P}+TBr_p(t)}$$

$$v_{Tel_{transcription}}(t) = \frac{k_{UbiqTel_P} \cdot UbiqTel_p(t)}{c_{UbiqTel_P}+UbiqTel_p(t)} \cdot \frac{k_{Tel_P} \cdot c_{Tel_P}}{c_{Tel_P}+Tel_p(t)} \cdot \frac{k_{HesC_P} \cdot c_{HesC_P}}{c_{HesC_P}+HesC_p(t)}$$

$$v_{Tgif_{transcription}}(t) = \frac{k_{Tgif_P} \cdot Tgif_p(t)}{c_{Tgif_P}+Tgif_p(t)} + \frac{k_{Ets1_P} \cdot Ets1_p(t)}{c_{Ets1_P}+Ets1_p(t)} + \frac{k_{Erg_P} \cdot Erg_p(t)}{c_{Erg_P}+Erg_p(t)} + \frac{k_{Hex_P} \cdot Hex_p(t)}{c_{Hex_P}+Hex_p(t)}$$

$$v_{VEGFR_{transcription}}(t) = \frac{k_{Alx1_P} \cdot Alx1_p(t)}{c_{Alx1_P}+Alx1_p(t)} + \frac{k_{Dri_P} \cdot Dri_p(t)}{c_{Dri_P}+Dri_p(t)} + \frac{k_{Ets1_P} \cdot Ets1_p(t)}{c_{Ets1_P}+Ets1_p(t)} + \frac{k_{Hex_P} \cdot Hex_p(t)}{c_{Hex_P}+Hex_p(t)}$$

$$v_{Wnt8_{transcription}}(t) = (\frac{k_{nBTCF_P} \cdot nBTCF_p(t)}{c_{nBTCF_P}+nBTCF_p(t)} + \frac{k_{Blimp1_P} \cdot Blimp1_p(t)}{c_{Blimp1_P}+Blimp1_p(t)}) \cdot \frac{k_{GroTCF_P} \cdot c_{GroTCF_P}}{c_{GroTCF_P}+GroTCF_p(t)}$$

$$v_{z13_{transcription}}(t) = \frac{k_{nBTCF_P} \cdot nBTCF_p(t)}{c_{nBTCF_P}+nBTCF_p(t)} \cdot \frac{k_{GroTCF_P} \cdot c_{GroTCF_P}}{c_{GroTCF_P}+GroTCF_p(t)} \cdot \frac{k_{Hnf6_P} \cdot c_{Hnf6_P}}{c_{Hnf6_P}+Hnf6_p(t)}$$

$$v_{UMADelta_{INPUT}}(t) = \frac{S1_{UMADelta} \cdot HillK_{UMADelta} \cdot t^{HillH_{UMADelta}}}{\theta_{1UMADelta}^{HillH_{UMADelta}+}t^{HillHUMADelta}} + S2_{UMADelta} \cdot HillK_{UMADelta} \cdot (1 - \frac{t^{HillH_{UMADelta}}}{theta2UMADelta^{HillH_{UMADelta}+}t^{HillH_{UMADelta}})})$$

$$v_{UMANrl_{INPUT}}(t) = \frac{S1_{UMANrl} \cdot HillK_{UMANrl} \cdot t^{HillH_{UMANrl}}}{\theta_{1UMANrl}^{HillH_{UMANrl}+}t^{HillHUMANrl}} + S2_{UMANrl} \cdot HillK_{UMANrl} \cdot (1 - \frac{t^{HillH_{UMANrl}}}{theta2UMANrl^{HillH_{UMANrl}+}t^{HillH_{UMANrl}})})$$

$$v_{Alx1_{degradation}}(t) = k_{deg_P} \cdot Alx1_p(t)$$

$$v_{Apobec_{degradation}}(t) = k_{deg_P} \cdot Apobec_p(t)$$

$$v_{Blimp1_{degradation}}(t) = k_{deg_P} \cdot Blimp1_p(t)$$

$$v_{Bra_{degradation}}(t) = k_{deg_P} \cdot Bra_p(t)$$
$$v_{Brn_{degradation}}(t) = k_{deg_P} \cdot Brn_p(t)$$
$$v_{CAPK_{degradation}}(t) = k_{deg_P} \cdot CAPK_p(t)$$
$$v_{CyP_{degradation}}(t) = k_{deg_P} \cdot CyP_p(t)$$
$$v_{Delta_{activation}}(t) = Delta2_p(t) \cdot Nrl_p(t) \cdot k_{Delta_{activation}}$$
$$v_{Delta_{degradation}}(t) = k_{deg_P} \cdot Delta_p(t)$$
$$v_{Delta_{inactivation}}(t) = Delta_p(t) \cdot k_{Delta_{inactivation}}$$
$$v_{Dpt_{degradation}}(t) = k_{deg_P} \cdot Dpt_p(t)$$
$$v_{Dri_{degradation}}(t) = k_{deg_P} \cdot Dri_p(t)$$
$$v_{ES_{degradation}}(t) = k_{deg_P} \cdot ES_p(t)$$
$$v_{Endo16_{degradation}}(t) = k_{deg_P} \cdot Endo16_p(t)$$
$$v_{Erg_{degradation}}(t) = k_{deg_P} \cdot Erg_p(t)$$
$$v_{Ets1_{degradation}}(t) = k_{deg_P} \cdot Ets1_p(t)$$
$$v_{Eve_{degradation}}(t) = k_{deg_P} \cdot Eve_p(t)$$
$$v_{Ficolin_{degradation}}(t) = k_{deg_P} \cdot Ficolin_p(t)$$
$$v_{FoxA_{degradation}}(t) = k_{deg_P} \cdot FoxA_p(t)$$
$$v_{FoxB_{degradation}}(t) = k_{deg_P} \cdot FoxB_p(t)$$
$$v_{FoxN23_{degradation}}(t) = k_{deg_P} \cdot FoxN23_p(t)$$
$$v_{FoxO_{degradation}}(t) = k_{deg_P} \cdot FoxO_p(t)$$
$$v_{FvMo_{degradation}}(t) = k_{deg_P} \cdot FvMo_p(t)$$
$$v_{GSK3i_{activation}}(t) = a_p(t)_{GSK3} \cdot a_p(t)_{frizzled} \cdot k_p(t)_{GSK3i_{activation}}$$
$$v_{GSK3i_{inactivation}}(t) = i_p(t)_{GSK3} \cdot k_p(t)_{GSK3i_{inactivation}}$$
$$v_{GataC_{degradation}}(t) = k_{deg_P} \cdot GataC_p(t)$$
$$v_{GataE_{degradation}}(t) = k_{deg_P} \cdot GataE_p(t)$$
$$v_{Gcad_{degradation}}(t) = k_{deg_P} \cdot Gcad_p(t)$$
$$v_{Gcm_{degradation}}(t) = k_{deg_P} \cdot Gcm_p(t)$$
$$v_{Gelsolin_{degradation}}(t) = k_{deg_P} \cdot Gelsolin_p(t)$$
$$v_{GroTCF_association}(t) = k_{GroTCF_{association}} \cdot Gro_p(t) \cdot TCF_p(t)$$
$$v_{GroTCF_{dissociation}}(t) = k_{GroTCF_{dissociation}} \cdot GroTCF_p(t)$$
$$v_{HesC_{degradation}}(t) = k_{deg_P} \cdot HesC_p(t)$$
$$v_{Hex_{degradation}}(t) = k_{deg_P} \cdot Hex_p(t)$$
$$v_{Hnf6_{degradation}}(t) = k_{deg_P} \cdot Hnf6_p(t)$$
$$v_{Hox_{degradation}}(t) = k_{deg_P} \cdot Hox_p(t)$$
$$v_{Kakapo_{degradation}}(t) = k_{deg_P} \cdot Kakapo_p(t)$$
$$v_{Lim_{degradation}}(t) = k_{deg_P} \cdot Lim_p(t)$$
$$v_{Msp130_{degradation}}(t) = k_{deg_P} \cdot Msp130_p(t)$$
$$v_{MspL_{degradation}}(t) = k_{deg_P} \cdot MspL_p(t)$$
$$v_{Not_{degradation}}(t) = k_{deg_P} \cdot Not_p(t)$$
$$v_{Notch_{activation}}(t) = Notch2_p(t) \cdot Delta2_p(t) \cdot k_{Notch_{activation}}$$

$$v_{Notch_{degradation}}(t) = k_{deg_P} \cdot Notch_p(t)$$
$$v_{Notch_{inactivation}}(t) = Notch_p(t) \cdot k_{Notch_{inactivation}}$$
$$v_{Nrl_{degradation}}(t) = k_{deg_P} \cdot Nrl_p(t)$$
$$v_{OrCt_{degradation}}(t) = k_{deg_P} \cdot OrCt_p(t)$$
$$v_{Otx_{degradation}}(t) = k_{deg_P} \cdot Otx_p(t)$$
$$v_{Pks_{degradation}}(t) = k_{deg_P} \cdot Pks_p(t)$$
$$v_{Pmar1_{degradation}}(t) = k_{deg_P} \cdot Pmar1_p(t)$$
$$v_{Sm27_{degradation}}(t) = k_{deg_P} \cdot Sm27_p(t)$$
$$v_{Sm30_{degradation}}(t) = k_{deg_P} \cdot Sm30_p(t)$$
$$v_{Sm50_{degradation}}(t) = k_{deg_P} \cdot Sm50_p(t)$$
$$v_{Snail_{degradation}}(t) = k_{deg_P} \cdot Snail_p(t)$$
$$v_{SoxB1_{degradation}}(t) = k_{deg_P} \cdot SoxB1_p(t)$$
$$v_{SoxC_{degradation}}(t) = k_{deg_P} \cdot SoxC_p(t)$$
$$v_{SuHN_{a}ssociation}(t) = k_{SuHN_{association}} \cdot Notch2_p(t) \cdot SuH_p(t)$$
$$v_{SuHN_{dissociation}}(t) = k_{SuHN_{dissociation}} \cdot SuHN_p(t)$$
$$v_{SuH_{degradation}}(t) = k_{deg_P} \cdot SuH_p(t)$$
$$v_{SuTx_{degradation}}(t) = k_{deg_P} \cdot SuTx_p(t)$$
$$v_{TBr_{degradation}}(t) = k_{deg_P} \cdot TBr_p(t)$$
$$v_{Tel_{degradation}}(t) = k_{deg_P} \cdot Tel_p(t)$$
$$v_{Tgif_{degradation}}(t) = k_{deg_P} \cdot Tgif_p(t)$$
$$v_{UMR_{degradation}}(t) = k_{deg_P} \cdot UMR_p(t)$$
$$v_{UVAOtx_{degradation}}(t) = k_{deg_P} \cdot UVAOtx_p(t)$$
$$v_{UbiqSoxB1_{degradation}}(t) = k_{deg_P} \cdot UbiqSoxB1_p(t)$$
$$v_{VEGFR_{degradation}}(t) = k_{deg_P} \cdot VEGFR_p(t)$$
$$v_{VEGFSignal_{a}ssociation}(t) = k_{VEGFSignal_{association}} \cdot L1_p(t) \cdot VEGFR_p(t) \cdot VEGF_p(t)$$
$$v_{VEGFSignal_{dissociation}}(t) = k_{VEGFSignal_{dissociation}} \cdot VEGFSignal_p(t)$$
$$v_{VEGF_{degradation}}(t) = k_{deg_P} \cdot VEGF_p(t)$$
$$v_{Wnt8_{degradation}}(t) = k_{deg_P} \cdot Wnt8_p(t)$$
$$v_{cBa_{degradation}}(t) = cB_p(t) \cdot a_p(t)_{GSK3} \cdot k_p(t)cB_{adeg}$$
$$v_{cB_{degradation}}(t) = k_{deg_P} \cdot cB_p(t)$$
$$v_{frizzleda_{activation}}(t) = i_p(t)_{frizzled} \cdot Wnt8_p(t) \cdot k_p(t)_{frizzleda_{activation}}$$
$$v_{frizzleda_{inactivation}}(t) = a_p(t)_{frizzled} \cdot k_p(t)_{frizzleda_{inactivation}}$$
$$v_{nBTCF_{a}ssociation}(t) = k_{nBTCF_{association}} \cdot cB_p(t) \cdot TCF_p(t)$$
$$v_{nBTCF_{dissociation}}(t) = k_{nBTCF_{dissociation}} \cdot nBTCF_p(t)$$
$$v_{z13_{degradation}}(t) = k_{deg_P} \cdot z13_p(t)$$
$$v_{UMADelta_{degradation}}(t) = k_{deg_P} \cdot UMADelta_p(t)$$
$$v_{UMANrl_{degradation}}(t) = k_{deg_P} \cdot UMANrl_p(t)$$
$$v_{L1_{degradation}}(t) = k_{deg_P} \cdot L1_p(t)$$
$$v_{UbiqAlx1_{degradation}}(t) = k_{deg_P} \cdot UbiqAlx1_p(t)$$

$$v_{UbiqES_{degradation}}(t) = k_{deg_P} \cdot UbiqES_p(t)$$
$$v_{UbiqEts1_{degradation}}(t) = k_{deg_P} \cdot UbiqEts1_p(t)$$
$$v_{UbiqHesC_{degradation}}(t) = k_{deg_P} \cdot UbiqHesC_p(t)$$
$$v_{UbiqHnf6_{degradation}}(t) = k_{deg_P} \cdot UbiqHnf6_p(t)$$
$$v_{UbiqSoxC_{degradation}}(t) = k_{deg_P} \cdot UbiqSoxC_p(t)$$
$$v_{UbiqTel_{degradation}}(t) = k_{deg_P} \cdot UbiqTel_p(t)$$
$$v_{Ets1_{INPUT}}(t) = \frac{S1_{Ets1} \cdot HillK_{Ets1} \cdot t^{HillH_{Ets1}}}{\theta_{1Ets1}^{HillH_{Ets1}+t^{HillHEts1}}} + S2_{Ets1} \cdot HillK_{Ets1} \cdot (1 - \frac{t^{HillH_{Ets1}}}{theta2Ets1^{HillH_{Ets1}+t^{HillH_{Ets1})}}})$$
$$v_{L1_{INPUT}}(t) = \frac{S1_{L1} \cdot HillK_{L1} \cdot t^{HillH_{L1}}}{\theta_{1L1}^{HillH_{L1}+t^{HillHL1}}} + S2_{L1} \cdot HillK_{L1} \cdot (1 - \frac{t^{HillH_{L1}}}{theta2L1^{HillH_{L1}+t^{HillH_{L1})}}})$$
$$v_{UbiqAlx1_{INPUT}}(t) = \frac{S1_{UbiqAlx1} \cdot HillK_{UbiqAlx1} \cdot t^{HillH_{UbiqAlx1}}}{\theta_{1UbiqAlx1}^{HillH_{UbiqAlx1}+t^{HillHUbiqAlx1}}} + S2_{UbiqAlx1} \cdot HillK_{UbiqAlx1} \cdot (1 - \frac{t^{HillH_{UbiqAlx1}}}{theta2UbiqAlx1^{HillH_{UbiqAlx1}+t^{HillH_{UbiqAlx1})}}})$$
$$v_{UbiqES_{INPUT}}(t) = \frac{S1_{UbiqES} \cdot HillK_{UbiqES} \cdot t^{HillH_{UbiqES}}}{\theta_{1UbiqES}^{HillH_{UbiqES}+t^{HillHUbiqES}}} + S2_{UbiqES} \cdot HillK_{UbiqES} \cdot (1 - \frac{t^{HillH_{UbiqES}}}{theta2UbiqES^{HillH_{UbiqES}+t^{HillH_{UbiqES})}}})$$
$$v_{UbiqEts1_{INPUT}}(t) = \frac{S1_{UbiqEts1} \cdot HillK_{UbiqEts1} \cdot t^{HillH_{UbiqEts1}}}{\theta_{1UbiqEts1}^{HillH_{UbiqEts1}+t^{HillHUbiqEts1}}} + S2_{UbiqEts1} \cdot HillK_{UbiqEts1} \cdot (1 - \frac{t^{HillH_{UbiqEts1}}}{theta2UbiqEts1^{HillH_{UbiqEts1}+t^{HillH_{UbiqEts1})}}})$$
$$v_{UbiqHesC_{INPUT}}(t) = \frac{S1_{UbiqHesC} \cdot HillK_{UbiqHesC} \cdot t^{HillH_{UbiqHesC}}}{\theta_{1UbiqHesC}^{HillH_{UbiqHesC}+t^{HillHUbiqHesC}}} + S2_{UbiqHesC} \cdot HillK_{UbiqHesC} \cdot (1 - \frac{t^{HillH_{UbiqHesC}}}{theta2UbiqHesC^{HillH_{UbiqHesC}+t^{HillH_{UbiqHesC})}}})$$
$$v_{UbiqHnf6_{INPUT}}(t) = \frac{S1_{UbiqHnf6} \cdot HillK_{UbiqHnf6} \cdot t^{HillH_{UbiqHnf6}}}{\theta_{1UbiqHnf6}^{HillH_{UbiqHnf6}+t^{HillHUbiqHnf6}}} + S2_{UbiqHnf6} \cdot HillK_{UbiqHnf6} \cdot (1 - \frac{t^{HillH_{UbiqHnf6}}}{theta2UbiqHnf6^{HillH_{UbiqHnf6}+t^{HillH_{UbiqHnf6})}}})$$
$$v_{UbiqSoxC_{INPUT}}(t) = \frac{S1_{UbiqSoxC} \cdot HillK_{UbiqSoxC} \cdot t^{HillH_{UbiqSoxC}}}{\theta_{1UbiqSoxC}^{HillH_{UbiqSoxC}+t^{HillHUbiqSoxC}}} + S2_{UbiqSoxC} \cdot HillK_{UbiqSoxC} \cdot (1 - \frac{t^{HillH_{UbiqSoxC}}}{theta2UbiqSoxC^{HillH_{UbiqSoxC}+t^{HillH_{UbiqSoxC})}}})$$
$$v_{UbiqTel_{INPUT}}(t) = \frac{S1_{UbiqTel} \cdot HillK_{UbiqTel} \cdot t^{HillH_{UbiqTel}}}{\theta_{1UbiqTel}^{HillH_{UbiqTel}+t^{HillHUbiqTel}}} + S2_{UbiqTel} \cdot HillK_{UbiqTel} \cdot (1 - \frac{t^{HillH_{UbiqTel}}}{theta2UbiqTel^{HillH_{UbiqTel}+t^{HillH_{UbiqTel})}}})$$
$$v_{Alx1_{degradation_M}}(t) = k_{deg_M} \cdot Alx1_M(t)$$
$$v_{Alx1_{translation}}(t) = translation_k \cdot Apobec_M(t)$$
$$v_{Apobec_{degradation_M}}(t) = k_{deg_M} \cdot Blimp1_M(t)$$
$$v_{Apobec_{translation}}(t) = translation_k \cdot Bra_M(t)$$
$$v_{Blimp1_{degradation_M}}(t) = k_{deg_M} \cdot Brn_M(t)$$
$$v_{Blimp1_{translation}}(t) = translation_k \cdot CAPK_M(t)$$
$$v_{Bra_{degradation_M}}(t) = k_{deg_M} \cdot CyP_M(t)$$
$$v_{Bra_{translation}}(t) = translation_k \cdot Delta_M(t)$$
$$v_{Brn_{degradation_M}}(t) = k_{deg_M} \cdot Dpt_M(t)$$
$$v_{Brn_{translation}}(t) = translation_k \cdot Dri_M(t)$$
$$v_{CAPK_{degradation_M}}(t) = k_{deg_M} \cdot ES_M(t)$$
$$v_{CAPK_{translation}}(t) = translation_k \cdot Endo16_M(t)$$
$$v_{CyP_{degradation_M}}(t) = k_{deg_M} \cdot Erg_M(t)$$
$$v_{CyP_{translation}}(t) = translation_k \cdot Ets1_M(t)$$
$$v_{Delta_{degradation_M}}(t) = k_{deg_M} \cdot Eve_M(t)$$
$$v_{Delta_{translation}}(t) = translation_k \cdot Ficolin_M(t)$$

$$v_{Dpt_{degradation_M}}(t) = k_{deg_M} \cdot FoxA_M(t)$$
$$v_{Dpt_{translation}}(t) = translation_k \cdot FoxB_M(t)$$
$$v_{Dri_{degradation_M}}(t) = k_{deg_M} \cdot FoxN23_M(t)$$
$$v_{Dri_{translation}}(t) = translation_k \cdot FoxO_M(t)$$
$$v_{ES_{degradation_M}}(t) = k_{deg_M} \cdot FvMo_M(t)$$
$$v_{ES_{translation}}(t) = translation_k \cdot GataC_M(t)$$
$$v_{Endo16_{degradation_M}}(t) = k_{deg_M} \cdot GataE_M(t)$$
$$v_{Endo16_{translation}}(t) = translation_k \cdot Gcad_M(t)$$
$$v_{Erg_{degradation_M}}(t) = k_{deg_M} \cdot Gcm_M(t)$$
$$v_{Erg_{translation}}(t) = translation_k \cdot Gelsolin_M(t)$$
$$v_{Ets1_{degradation_M}}(t) = k_{deg_M} \cdot HesC_M(t)$$
$$v_{Ets1_{translation}}(t) = translation_k \cdot Hex_M(t)$$
$$v_{Eve_{degradation_M}}(t) = k_{deg_M} \cdot Hnf6_M(t)$$
$$v_{Eve_{translation}}(t) = translation_k \cdot Hox_M(t)$$
$$v_{Ficolin_{degradation_M}}(t) = k_{deg_M} \cdot Kakapo_M(t)$$
$$v_{Ficolin_{translation}}(t) = translation_k \cdot Lim_M(t)$$
$$v_{FoxA_{degradation_M}}(t) = k_{deg_M} \cdot Msp130_M(t)$$
$$v_{FoxA_{translation}}(t) = translation_k \cdot MspL_M(t)$$
$$v_{FoxB_{degradation_M}}(t) = k_{deg_M} \cdot Not_M(t)$$
$$v_{FoxB_{translation}}(t) = translation_k \cdot Notch_M(t)$$
$$v_{FoxN23_{degradation_M}}(t) = k_{deg_M} \cdot Nrl_M(t)$$
$$v_{FoxN23_{translation}}(t) = translation_k \cdot OrCt_M(t)$$
$$v_{FoxO_{degradation_M}}(t) = k_{deg_M} \cdot Otx_M(t)$$
$$v_{FoxO_{translation}}(t) = translation_k \cdot Pks_M(t)$$
$$v_{FvMo_{degradation_M}}(t) = k_{deg_M} \cdot Pmar1_M(t)$$
$$v_{FvMo_{translation}}(t) = translation_k \cdot Sm27_M(t)$$
$$v_{GataC_{degradation_M}}(t) = k_{deg_M} \cdot Sm30_M(t)$$
$$v_{GataC_{translation}}(t) = translation_k \cdot Sm50_M(t)$$
$$v_{GataE_{degradation_M}}(t) = k_{deg_M} \cdot Snail_M(t)$$
$$v_{GataE_{translation}}(t) = translation_k \cdot SoxB1_M(t)$$
$$v_{Gcad_{degradation_M}}(t) = k_{deg_M} \cdot SoxC_M(t)$$
$$v_{Gcad_{translation}}(t) = translation_k \cdot SuH_M(t)$$
$$v_{Gcm_{degradation_M}}(t) = k_{deg_M} \cdot SuTx_M(t)$$
$$v_{Gcm_{translation}}(t) = translation_k \cdot TBr_M(t)$$
$$v_{Gelsolin_{degradation_M}}(t) = k_{deg_M} \cdot Tel_M(t)$$
$$v_{Gelsolin_{translation}}(t) = translation_k \cdot Tgif_M(t)$$
$$v_{HesC_{degradation_M}}(t) = k_{deg_M} \cdot UMR_M(t)$$
$$v_{HesC_{translation}}(t) = translation_k \cdot UVAOtx_M(t)$$

$$v_{Hex_{degradation_M}}(t) = k_{deg_M} \cdot UbiqSoxB1_M(t)$$
$$v_{Hex_{translation}}(t) = translation_k \cdot VEGFR_M(t)$$
$$v_{Hnf6_{degradation_M}}(t) = k_{deg_M} \cdot VEGF_M(t)$$
$$v_{Hnf6_{translation}}(t) = translation_k \cdot Wnt8_M(t)$$
$$v_{Hox_{degradation_M}}(t) = k_{deg_M} \cdot cB_M(t)$$
$$v_{Hox_{translation}}(t) = translation_k \cdot z13_M(t)$$
$$v_{Kakapo_{degradation_M}}(t) = k_{deg_M} \cdot Alx1_M(t)$$
$$v_{Kakapo_{translation}}(t) = translation_k \cdot Apobec_M(t)$$
$$v_{Lim_{degradation_M}}(t) = k_{deg_M} \cdot Blimp1_M(t)$$
$$v_{Lim_{translation}}(t) = translation_k \cdot Bra_M(t)$$
$$v_{Msp130_{degradation_M}}(t) = k_{deg_M} \cdot Brn_M(t)$$
$$v_{Msp130_{translation}}(t) = translation_k \cdot CAPK_M(t)$$
$$v_{MspL_{degradation_M}}(t) = k_{deg_M} \cdot CyP_M(t)$$
$$v_{MspL_{translation}}(t) = translation_k \cdot Delta_M(t)$$
$$v_{Not_{degradation_M}}(t) = k_{deg_M} \cdot Dpt_M(t)$$
$$v_{Not_{translation}}(t) = translation_k \cdot Dri_M(t)$$
$$v_{Notch_{degradation_M}}(t) = k_{deg_M} \cdot Endo16_M(t)$$
$$v_{Notch_{translation}}(t) = translation_k \cdot Erg_M(t)$$
$$v_{Nrl_{degradation_M}}(t) = k_{deg_M} \cdot Ets1_M(t)$$
$$v_{Nrl_{translation}}(t) = translation_k \cdot Eve_M(t)$$
$$v_{OrCt_{degradation_M}}(t) = k_{deg_M} \cdot Ficolin_M(t)$$
$$v_{OrCt_{translation}}(t) = translation_k \cdot FoxA_M(t)$$
$$v_{Otx_{degradation_M}}(t) = k_{deg_M} \cdot FoxB_M(t)$$
$$v_{Otx_{translation}}(t) = translation_k \cdot FoxN23_M(t)$$
$$v_{Pks_{degradation_M}}(t) = k_{deg_M} \cdot FoxO_M(t)$$
$$v_{Pks_{translation}}(t) = translation_k \cdot FvMo_M(t)$$
$$v_{Pmar1_{degradation_M}}(t) = k_{deg_M} \cdot GataC_M(t)$$
$$v_{Pmar1_{translation}}(t) = translation_k \cdot GataE_M(t)$$
$$v_{Sm27_{degradation_M}}(t) = k_{deg_M} \cdot Gcad_M(t)$$
$$v_{Sm27_{translation}}(t) = translation_k \cdot Gcm_M(t)$$
$$v_{Sm30_{degradation_M}}(t) = k_{deg_M} \cdot Gelsolin_M(t)$$
$$v_{Sm30_{translation}}(t) = translation_k \cdot HesC_M(t)$$
$$v_{Sm50_{degradation_M}}(t) = k_{deg_M} \cdot Hex_M(t)$$
$$v_{Sm50_{translation}}(t) = translation_k \cdot Hnf6_M(t)$$
$$v_{Snail_{degradation_M}}(t) = k_{deg_M} \cdot Hox_M(t)$$
$$v_{Snail_{translation}}(t) = translation_k \cdot Kakapo_M(t)$$
$$v_{SoxB1_{degradation_M}}(t) = k_{deg_M} \cdot Lim_M(t)$$
$$v_{SoxB1_{translation}}(t) = translation_k \cdot Msp130_M(t)$$

$$v_{SoxC_{degradation_M}}(t) = k_{deg_M} \cdot MspL_M(t)$$
$$v_{SoxC_{translation}}(t) = translation_k \cdot Not_M(t)$$
$$v_{SuH_{degradation_M}}(t) = k_{deg_M} \cdot Notch_M(t)$$
$$v_{SuH_{translation}}(t) = translation_k \cdot Nrl_M(t)$$
$$v_{SuTx_{degradation_M}}(t) = k_{deg_M} \cdot OrCt_M(t)$$
$$v_{SuTx_{translation}}(t) = translation_k \cdot Otx_M(t)$$
$$v_{TBr_{degradation_M}}(t) = k_{deg_M} \cdot Pks_M(t)$$
$$v_{TBr_{translation}}(t) = translation_k \cdot Pmar1_M(t)$$
$$v_{Tel_{degradation_M}}(t) = k_{deg_M} \cdot Sm27_M(t)$$
$$v_{Tel_{translation}}(t) = translation_k \cdot Sm30_M(t)$$
$$v_{Tgif_{degradation_M}}(t) = k_{deg_M} \cdot Sm50_M(t)$$
$$v_{Tgif_{translation}}(t) = translation_k \cdot Snail_M(t)$$
$$v_{UMR_{degradation_M}}(t) = k_{deg_M} \cdot SoxB1_M(t)$$
$$v_{UMR_{translation}}(t) = translation_k \cdot SoxC_M(t)$$
$$v_{UVAOtx_{degradation_M}}(t) = k_{deg_M} \cdot SuH_M(t)$$
$$v_{UVAOtx_{translation}}(t) = translation_k \cdot SuTx_M(t)$$
$$v_{UbiqSoxB1_{degradation_M}}(t) = k_{deg_M} \cdot TBr_M(t)$$
$$v_{UbiqSoxB1_{translation}}(t) = translation_k \cdot Tel_M(t)$$
$$v_{VEGFR_{degradation_M}}(t) = k_{deg_M} \cdot Tgif_M(t)$$
$$v_{VEGFR_{translation}}(t) = translation_k \cdot UMADelta_M(t)$$
$$v_{VEGF_{degradation_M}}(t) = k_{deg_M} \cdot UMANrl_M(t)$$
$$v_{VEGF_{translation}}(t) = translation_k \cdot UMR_M(t)$$
$$v_{Wnt8_{degradation_M}}(t) = k_{deg_M} \cdot UbiqSoxB1_M(t)$$
$$v_{Wnt8_{translation}}(t) = translation_k \cdot VEGFR_M(t)$$
$$v_{cB_{degradation_M}}(t) = k_{deg_M} \cdot Wnt8_M(t)$$
$$v_{cB_{translation}}(t) = translation_k \cdot cB_M(t)$$
$$v_{z13_{degradation_M}}(t) = k_{deg_M} \cdot z13_M(t)$$
$$v_{z13_{translation}}(t) = translation_k \cdot Alx1_M(t)$$
