# Supplementary material for: Monte Carlo analysis of an ODE Model of the Sea Urchin Endomesoderm Network
Source: BMC Syst Biol. 2009 Aug 23;3:83. doi: 10.1186/1752-0509-3-83 (PMC2739852; doi:10.1186/1752-0509-3-83)
Supplement: Additional file 6 — SBML Model. Model in SBML format. Parameter values are set to arbitrary values. Also available on BioModels under ID MODEL2133240427. [file 1752-0509-3-83-S6.pdf]

Suplemental Filr 6 for 'Monte Carlo analysis of an ODE Model of the Sea Urchin Endomesoderm Network': Robustness to Random Parameter Variations

Clemens Kühn[1,2], Christoph Wierling[1] , Alexander Kühn[1] , Edda Klipp[2], Georgia Panopoulou[1], Hans Lehrach[1], Albert J. Poustka*[1]

[1] Max-Planck-Institute for Molecular Genetics, Ihnestr 63-73, 14195 Berlin, Germany
[2] Humboldt Universität zu Berlin, Institute for Biology, Invalidenstr 42, 10115 Berlin, Germany

Email: Clemens Kühn - clemens.kuehn@biologie.hu-berlin.de; Christoph Wierling - wierling@molgen.mpg.de; Alexander Kühn - kuehn_a@molgen.mpg.de; Edda Klipp - edda.klipp@biologie.hu-belrin.de; Georgia Panopoulou - panopoul@molgen.mpg.de; Hans Lehrach - lehrach@molgen.mpg.de; Albert J. Poustka - poustka@molgen.mpg.de;

*Corresponding author

Result

Based on the analysis proposed in the article, we computed the robustness of expression of different genes against random parameter variations using Monte Carlo simulations.

To this end, we performed multiple simulation runs with different parameter sets, each generating a distinct result. The resulting mRNA concentrations have been averaged for each time point, also recording the variation. Using the coefficient of variation $c_v = \sigma/\mu$, the variation in expression of different genes can be compared. It indicates whether the expression of a gene is highly affected by or robust against parameter changes.

The robustness computed here is not synonymous with robustness as commonly used in biological contexts [1]. In most publications, robustness in a GRN implies that the main features of the system are preserved when small deviations from some natural condition occur (e.g. small changes in individual parameter values). In contrast, we simulate the model using sets of arbitrary parameter values, which might be well out of range of biologically meaningful values.

We computed c_v for all genes in the model using 800 parameter sets under unperturbed conditions. For each gene, the highest c_v (pertaining to the lowest robustness) of all time points is used as an indicator of the genes robustness. Generally, the robustness is higher at earlier measurement points. This is due to

variations in expression of genes upstream of the analyzed genes that take time to propagate to the affected gene.

Table 1 gives an overview over the c_v of each gene in the network. The c_v range from 0.21 to 25.69 for the 800 sampled parameter sets used, indicating that the genes in the network, as it is, differ substantially in their robustness against random parameter changes.

We could neither detect a correlation between node in-degree (the number of regulatory inputs to a particular gene) and robustness nor could we detect a correlation between node out-degree (the number of genes a particular gene regulates) and robustness. The first correlation would indicate that the robustness of expression increases with increased number of regulators, the second would indicate that important regulatory genes are more robust than other genes. We could also not find a correlation between a gene's position in the network (early endomeso, early PMC, late endoderm and mesoderm, late PMC) and its robustness.

The genes of the network that inhibit their own expression, namely *SoxB1*, *GataC*, *FoxB*, *TBr*, *FoxA* and *Blimp1* are the genes with the lowest c_v. This indicates that autoinhibition strongly stabilizes a genes expression. Interestingly, the next five genes in the list (*Eve*, *Apobec*, *OrCt*, *GataE*, *HesC*) all have at least one negative regulatory input.

Besides the robustness of negatively regulated genes, the results indicate that the current architecture does not favor any specific set of genes in their robustness against random parameter changes, that the network is too small to detect significant sets of genes that are expressed with similar robustness or that the parameter values used in the analysis are not in the range in which the system can produce a robust response.

Method

In order to infer the robustness of different components of the Endomesoderm Network model we employed simulation results of the unperturbed model M_0. Using a number of parameter sets J, we obtained different concentrations $s(k, 0, j, t)$ for each parameter set j in J. For each time point, we computed the mean μ and standard deviation σ^2 assuming that the simulation results are normally distributed around a true concentration value. The relative variation $c_v = \sigma/\mu$ provides a comparable measure of variation in relation to the specific mean. We used this relative variation here because expression strength among the genes of the models differs substantially. The relative variation is an indicator of how much parameter values affect the expression of different genes, in other words, how robust the genes are to changes in

parameters. To infer an overall robustness of each gene for the entire simulation time, we considered the highest relative variation (lowest robustness) as a lower bound.

Table 1. Robustness to random parameter changes.

Coefficient of variation (c_v) as a means of robustness against parameter changes, node indegree (ID) and outdegree (OD) for all genes in the Endomesoderm GRN.

Gene	c_v	ID	OD	Gene	c_v	ID	OD
SoxB1	0.211	3	1	Sm27	2.632	7	0
GataC	0.291	4	1	MspL	2.685	5	0
FoxB	0.404	6	2	VEGFR	2.737	4	1
Tbr	0.49	3	5	Ficolin	2.759	4	0
FoxA	0.695	6	2	Pks	2.778	3	0
Blimp1	0.698	7	4	SuTx	2.797	2	0
Eve	0.81	3	2	FvMo	2.867	2	0
Apobec	0.864	2	0	Dpt	2.927	2	0
OrCt	0.994	2	0	Snail	2.945	1	1
GataE	1.116	3	4	Dri	2.99	2	6
HesC	1.187	2	5	Endo16	2.992	2	0
Otx	1.282	4	7	Kakapo	3.773	1	0
Nrl	2.153	7	1	Gelsolin	3.904	1	0
Brn	2.388	1	3	Gcm	5.205	7	7
Not	2.399	1	0	Wnt8	5.245	3	1
Hnf6	2.443	1	4	Ets1	5.44	2	13
Sm50	2.49	8	0	Pmar1	7.09	2	1
Msp130	2.586	8	0	Bra	7.847	3	9
Lim	2.586	2	0	Hox	9.613	4	4
CAPK	2.595	1	0	CyP	10.28	2	0
Sm30	2.599	1	0	Delta	10.73	3	1
				Alx1	25.70	5	7

References

1. Kitano H: **Biologial robustness**. *Nature Reviews Genetics* 2004, **5**:826–837.
